# Predictors for survival in patients with Alzheimer’s disease: a large comprehensive meta-analysis

**DOI:** 10.1038/s41398-024-02897-w

**Published:** 2024-04-10

**Authors:** Xiaoting Zheng, Shichan Wang, Jingxuan Huang, Chunyu Li, Huifang Shang

**Affiliations:** Department of Neurology, Laboratory of Neurodegenerative Disorders, National Clinical Research Center for Geriatrics, West China Hospital, Sichuan University, Chengdu, 610041 China

**Keywords:** Diseases, Neuroscience

## Abstract

The prevalence of Alzheimer’s disease (AD) is increasing as the population ages, and patients with AD have a poor prognosis. However, knowledge on factors for predicting the survival of AD remains sparse. Here, we aimed to systematically explore predictors of AD survival. We searched the PubMed, Embase and Cochrane databases for relevant literature from inception to December 2022. Cohort and case-control studies were selected, and multivariable adjusted relative risks (RRs) were pooled by random-effects models. A total of 40,784 reports were identified, among which 64 studies involving 297,279 AD patients were included in the meta-analysis after filtering based on predetermined criteria. Four aspects, including demographic features (*n* = 7), clinical features or comorbidities (*n* = 13), rating scales (*n* = 3) and biomarkers (*n* = 3), were explored and 26 probable prognostic factors were finally investigated for AD survival. We observed that AD patients who had hyperlipidaemia (RR: 0.69) were at a lower risk of death. In contrast, male sex (RR: 1.53), movement disorders (including extrapyramidal signs) (RR: 1.60) and cancer (RR: 2.07) were detrimental to AD patient survival. However, our results did not support the involvement of education, hypertension, APOE genotype, Aβ_42_ and t-tau in AD survival. Our study comprehensively summarized risk factors affecting survival in patients with AD, provided a better understanding on the role of different factors in the survival of AD from four dimensions, and paved the way for further research.

## Introduction

Alzheimer’s disease (AD) is a chronic neurodegenerative disorder with progressive cognitive impairment and is the predominant form of dementia [1, 2]. As of 2020, approximately 55 million people worldwide are living with dementia, and that number is predicted to reach 78 million by 2030 [3]. The mortality of AD increased by 29.28% from 1990 to 2019 with the increase in the aging population [4, 5]. Moreover, AD and other dementias were the fourth cause of disability-adjusted life-years (DALYs) for those aged 75 years and older [5], leading to a tremendous burden on society and caregivers.

In the context of emerging treatments for preclinical AD, despite intensive research and development efforts to identify therapeutic drugs, there is still no effective strategy to stop progression due to insufficient knowledge of the etiology of AD [6]. Under these circumstances, focusing on influential factors potentiating AD progression since diagnosis is critical for neurologists and patients’ families. Great efforts have been made [7–10] to determine predictive factors for survival of AD, and a number of predictors that may worsen the disease prognosis have been identified. Prognostic factors such as age at diagnosis, underweight, extrapyramidal signs (EPS) and psychosis, and history of vascular or heart disease appear to be key players in the progression of AD [7, 8, 10–14]. Nutritional status was found to be the exact predictor of an unfavorable course, which was suggested to therefore form part of the clinical evaluation [15]. One study proposed that combination therapies targeting AD pathophysiology and vascular risk factors might enhance therapeutic effects [16]. Patients could benefit similarly from remedies that target modifiable factors. However, previous studies which tried to sum up the predictors only kept eyes on a limited dimension of factors, and on account of the research inconsistencies and limited number of studies, the conclusions were inauthentic and the supportive reasons were inadequate [7, 17, 18]. Therefore, an extensive summary is urgently needed, and we performed this meta-analysis to fill this research gap.

With the aim of further understanding the prognostic factors of AD and guiding clinical work from specifically modifiable issues, we designed a systematic meta-analysis to summarize predictive factors for the survival and quality of life of AD patients from various dimensions.

## Methods

### Search strategy

The PubMed, Embase and Cochrane databases were systematically searched from inception to December 2022 by terms “Alzheimer disease OR dementia OR Alzheimer* OR AD OR Dement*” AND “prognosis* OR progress* OR survival OR outcome OR mortality OR death OR hazard” by two independent researchers (XZ and SW). Furthermore, we refined the search scope for case-control or cohort studies by limiting “prospective OR retrospective OR cohort OR case-control OR case control OR consecutive” in the title or abstract. The comprehensive meta-analysis was performed following the Preferred Reporting Item for Systematic Review and Meta-analysis (2020) guidelines [19] (Supplementary Table S1). The titles and abstracts of all retrieved articles were reviewed. We also considered other publications in the full-read reports reference lists as supplementary papers. There were no restrictions applied in the literature search. The protocol for the study was registered with PROSPERO (registration number: CRD42022365357).

### Selection criteria

The inclusion criteria were as follows: 1) the diagnostic criteria for AD patients were clearly stated; 2) case-control or cohort studies published in English; 3) the literature reported risk factors for the survival outcome of AD; and 4) the study provided adjusted effect sizes, relative risks (RRs), or hazard ratios (HRs) with 95% confidence intervals (CIs) through multivariate analysis. The exclusion criteria were as follows: 1) duplicate literature without new data; 2) incomplete data or odds ratios (ORs) as effect variables; 3) case reports, conference abstracts, reviews, comments, author replies and editorial materials; 4) studies on animals, cells and genes; and 5) patients diagnosed with any other type of dementia, including but not limited to vascular dementia, frontotemporal dementia, Parkinson’s disease dementia, and dementia with Lewy bodies. Further, considering the search scope of observational studies, we excluded predictors involving only treatment or nursing care to avoid inadequate aggregation.

### Data extraction and quality assessment

Data extraction was performed by two independent researchers (XZ and SW). If a study had multiple estimates for the same factor, we only selected the estimates with the most adjusted variables and the longest follow-up time. According to previous survival research, survival was defined as the time when instruments were needed to sustain vital signs or mortality data retrieved from the registration system. For several causes of death, all-cause mortality was chosen to avoid underestimating the real death toll. Only when there were enough studies to conduct meta-analysis (≥3 studies concerning a potential variable) could the adjusted results be extracted. In addition, different studies might use different models or classifications of factors concerning survival and report various estimates in terms of one reference. Given that, we combined the poly-values into an overall value by a random-effects model. For variable inclusion, only categorical data providing the same classification criteria and continuous variables (per year/point increase) were included. The author, publication year, sample size, country, AD diagnostic criteria, included factors, endpoints, mean age, sex ratio, follow-up period, mean disease duration and median survival time were listed.

In addition, we exhibited the Newcastle-Ottawa Scale (NOS) and confounding factors of each eligible study. The endpoints included death, institutionalization, nursing home place (NHP) and cognitive decline (especially rapid decline). For possible factors and endpoints, the combined estimates of items were extracted from four aspects, and the details are described in the Supplementary Materials (Supplementary Table S2). All the variables of rating scales, clinical features or comorbidities were considered at diagnosis or at enrollment. Quality assessment was performed by two independent researchers (XZ and SW) using NOS scores. When there was divergence between the other two researchers, a third researcher was consulted to help reach a consensus.

### Statistical analysis

To assess the potential impact on survival, the RR with a 95% CI was used as the estimate to be pooled for quantitative synthesis. Due to the adjusted survival time, the HR was considered equal to the RR for analysis, while studies that reported ORs were excluded for their tendency to overestimate the effect size. Four aspects were investigated to probe factors influencing the survival of AD. The primary outcome was the combined adjusted RR and 95% CI for mortality. Additionally, we consolidated the remaining endpoints, including institutionalization, NHP and cognitive decline, into “poor prognosis” as the secondary outcome to represent quality of life.

The multivariable-adjusted estimates and 95% CIs were transformed into log relative risks to calculate combined values using the random-effects model. As a result, those whose effect value was the same as the lower and upper CIs were eliminated to obtain a calculable standard error (SE). For those that provided values with opposite reference objects, we converted them into a unified one to achieve consistency [20].

Heterogeneity was assessed using the Q test and quantified by the I² metric. I² < 25% indicated no evidence of heterogeneity; 25% < I² < 50% indicated acceptable heterogeneity, and in such cases, the fixed-effects model was adopted for pooled analysis; 50% < I² < 75% indicated possible heterogeneity; and I² > 75% indicated considerable heterogeneity, for which the random-effects model was chosen and further analysis was performed. To explain and reduce heterogeneity, subgroup analysis was applied if necessary. In addition, for heterogeneity that could not be explained, a multivariate sensitivity analysis was performed to examine if the pooled effect size was influenced by sequentially omitting individual studies and to detect the stability of results as well. Meanwhile, a meta-regression (*n* ≥ 10) was performed to explore the potential source of heterogeneity using the conservative Hartung–Knapp method [21, 22] and to assess the underlying interaction of study characteristics, with the terms age, sex, geographic region, sample size, NOS scores and follow-up period. The Egger test was used to detect potential publication bias, and the trim-and-fill method was constructed for adjustment when significant bias was found.

All of the above statistical analyses were performed using Stata 15.1, with a two-tailed *p* < 0.05 considered indicative of statistical significance.

## Results

### Literature retrieval and characteristics

**Fig. 1:**
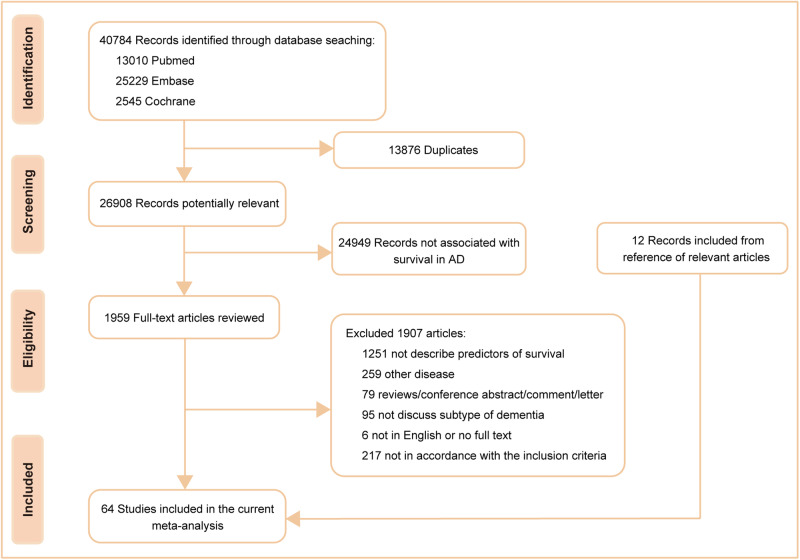
PRISMA flowchart for systematic review and meta-analysis. Flowchart of the literature search according to Preferred Reporting Item for Systematic Review and Meta-analysis (PRISMA).

According to the preset retrieval strategy, a total of 40,784 articles were considered from the outset. By excluding 13,876 duplicates and 24,949 records not associated with survival in AD, 1959 potential articles and an additional 12 from reference lists were fully reviewed. A further re-evaluation of each article led to the inclusion of 64 studies [11, 23–85] concerning 26 probable prognostic factors, which were categorized into four groups, namely, demographic features (*n* = 7), clinical features or comorbidities (*n* = 13), rating scales (*n* = 3) and biomarkers (*n* = 3). The detailed search flow diagram is shown in Fig. 1, and the statistically significant predictors are listed in Table 1. Moreover, the characteristics of the 64 eligible studies involving 297,279 AD patients are summarized in Supplementary Table S3. Confounding factors for the included studies are shown in Supplementary Table S4. The total results are shown in Supplementary Table S5 and the specific forest plots are listed in Supplementary Figs. S1–5.

**Table 1:** Meta-analysis of prognostic factors for mortality in patients with AD.

Prognostic factors	Number of studies	Pooled RR and 95% CI	*P* value	I^2^
Demographic features (4)				
Age (per year increase)	27	1.05 (1.04–1.07)	<0.001	92.2%
Age of onset (per year increase)	6	1.03 (1.01–1.05)	0.003	73.9%
Sex (ref: female)	37	1.58 (1.49–1.68)	<0.001	84.8%
Race (ref: none-white)	8	1.36 (1.21–1.53)	<0.001	75.8%
clinical features or comorbidities (10)				
Hyperlipidaemia	4	0.69 (0.59–0.80)	<0.001	0.0%
Cancer	3	2.07 (1.17–3.67)	0.013	92.9%
Movement disorders (including EPS)	7	1.60 (1.32–1.93)	<0.001	58.4%
NPS	14	1.16 (1.08–1.24)	<0.001	94.2%
Depression	7	1.12 (1.03–1.22)	0.011	26.4%
Heart disease	12	1.24 (1.11–1.37)	<0.001	76.7%
Cerebrovascular disease	12	1.30 (1.20–1.41)	<0.001	61.6%
Respiratory disease	5	1.23 (1.19–1.27)	<0.001	11.5%
Somatic comorbidity score	4	1.24 (1.06–1.44)	0.007	99.6%
Diabetes mellitus	12	1.30 (1.15–1.48)	<0.001	75.1%
Rating scales (3)				
MMSE scores (per point increase)	15	0.93 (0.91–0.95)	<0.001	85.6%
ADL scores (per point increase)	10	1.11 (1.07–1.16)	<0.001	93.4%
PSMS scores (per point increase)	4	1.09 (1.07–1.10)	<0.001	36.1%

*AD* Alzheimer’s disease, *RR* relative risk, *CI* confidence intervals, *MMSE* The Mini Mental State Examination, *ADL* Activity of Daily Living, *PSMS* Physical Self-Maintenance Scale, *EPS* extrapyramidal signs, *NPS* neuropsychiatric symptoms.

### Primary outcomes

#### Demographic features

**Fig. 2:**
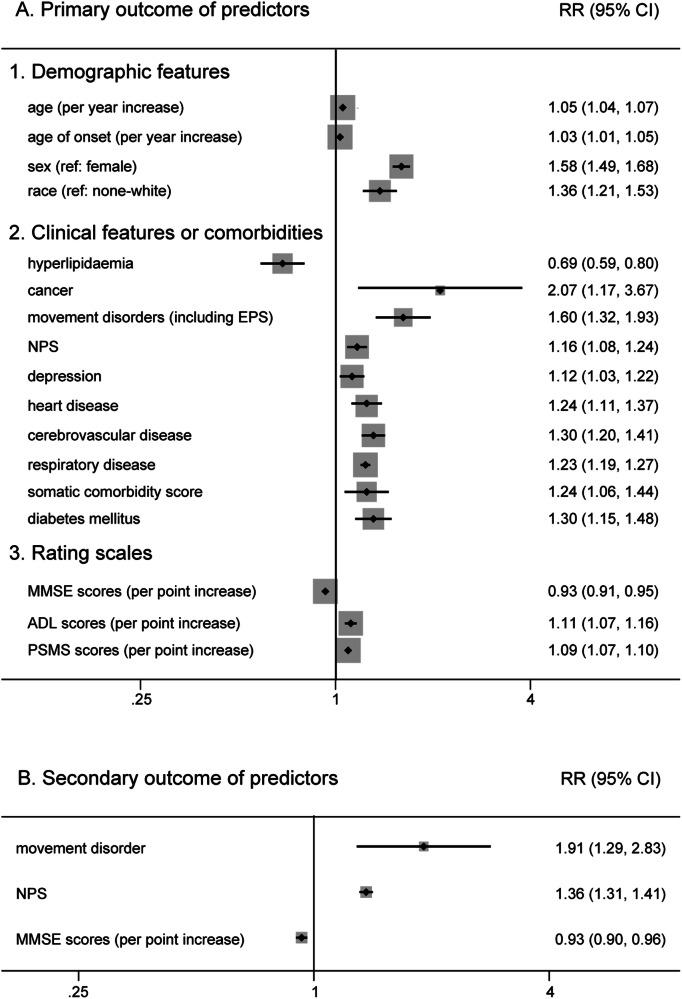
Forest plot of the prognostic factors in AD. The forest plot displays meta-analysis results of the prognostic factors in AD. AD Alzheimer’s disease, RR relative risk, CI confidence intervals.

Six factors (age, sex, race, years of education, marital status and smoking) for which there was prior evidence for an association with AD survival were included in the primary analysis (Supplementary Fig. S1). We found that there was a poor prognosis for older patients (RR 1.05, 95% CI 1.04–1.07 for baseline age; RR 1.03, 95% CI 1.01–1.05 for age of onset), males (RR 1.58, 95% CI 1.49–1.68) and white patients (RR 1.36, 95% CI 1.21–1.53) (Fig. 2). However, years of education (RR 1.00, 95% CI 0.98–1.02), living alone (RR 1.07, 95% CI 0.97–1.19), and smoking (RR 1.00, 95% CI 1.00–1.01) did not show a significant association.

#### Clinical features or comorbidities

Clinical features or comorbidities also play an important role in the prognosis of AD (Supplementary Fig. S2). In our analysis, those who had hyperlipidaemia (RR 0.69, 95% CI 0.59–0.80) had a lower risk of death (Fig. 2). In contrast, we found that manifestations of movement disorders (including EPS) (RR 1.60, 95% CI 1.32–1.93) and cancer (RR 2.07, 95% CI 1.17–3.67) were more detrimental to AD patient survival. Moreover, other features, such as neuropsychiatric symptoms (NPS) (RR 1.16, 95% CI 1.08–1.24), depression (RR 1.12, 95% CI 1.03–1.22), heart disease (RR 1.24, 95% CI 1.11–1.37), cerebrovascular disease (RR 1.30, 95% CI 1.20–1.41), respiratory disease (RR 1.23, 95% CI 1.19–1.27), diabetes mellitus (RR 1.30, 95% CI 1.15–1.48) and a higher somatic comorbidity score (RR 1.24, 95% CI 1.06–1.44), were associated with poor prognosis (Fig. 2). Beyond that, the pooled analysis failed to exhibit a significant outcome in patients with a history of hypertension (RR 1.19, 95% CI 1.00–1.41), wandering or falling (RR 1.39, 95% CI 0.95–2.06) and vascular risk factors (VRF) (RR 1.02, 95% CI 0.93–1.13).

#### Rating scales

For rating scales (Supplementary Fig. S3), patients with higher activity of daily living (ADL) scores (RR 1.11, 95% CI 1.07–1.16) and physical self-maintenance scale (PSMS) scores (RR 1.09, 95% CI 1.07–1.10), which indicated a lack of self-care ability, had an increased risk of death (Fig. 2). Similarly, higher Mini-Mental State Examination (MMSE) scores, which indicated relatively good cognitive function, decreased the risk for shorter survival (RR 0.93, 95% CI 0.91–0.95) (Fig. 2).

#### Biomarkers

An increasing number of studies have been devoted to elucidating the impact of biomarkers in developing AD rather than survival since diagnosis. However, due to the various cut-off values among different researches, it is not easy to perform quantitative analysis for all biomarkers. Therefore, only three biomarkers were analyzed in the current study (Supplementary Fig. S4). We found that neither apolipoprotein E (APOE) ε4 carriers (RR 0.94, 95% CI 0.78–1.14), the level of cerebrospinal fluid (CSF) β-amyloid (Aβ_42_) (RR 1.09, 95% CI 0.91–1.32) nor total tau protein (t-tau) (RR 1.00, 95% CI 1.00–1.01) had a significant impact on AD patient survival.

### Secondary outcome

In the secondary analysis, nine potential factors were calculated (Supplementary Fig. S5). We found that movement disorders (including EPS) (RR 1.76, 95% CI 1.11–2.79) and NPS (RR 1.35, 95% CI 1.25–1.46) had a detrimental influence on the prognosis of AD. Same as before, higher MMSE scores (RR 0.93, 95% CI 0.90–0.96) were associated with longer survival (Fig. 2). However, age (RR 1.02, 95% CI 0.98–1.06 for baseline age; RR 0.98, 95% CI 0.92–1.05 for age of onset), male sex (RR 0.92, 95% CI 0.81–1.04), living alone (RR 1.67, 95% CI 0.66–4.26), increased ADL scores (RR 1.05, 95% CI 0.96–1.16) and APOE ε4 carrier (RR 0.93, 95% CI 0.72–1.19) had no evident effect on living quality (institutionalization, NHP and cognitive decline) in patients with AD.

### Heterogeneity and sensitivity analysis

Heterogeneity exists in some combination of this meta-analysis. In the primary outcome, depression (I^2^ = 26.4%), respiratory disease (I^2^ = 11.5%), hyperlipidaemia (I^2^ < 0.001) and PSMS scores (I^2^ = 36.1%) demonstrated unobvious or acceptable heterogeneity. We found that the heterogeneity of movement disorders was reduced by removing the study performed by Stern et al. [37]. Meanwhile, subgroup analysis was performed to reveal possible heterogeneity among studies, and the heterogeneity for age of onset, sex, cancer, NPS (subdivided into four types: behavioral problems, specific hallucinations or delusions, psychosis, mood disorder, any of the above symptoms), cerebrovascular disease, heart disease (cardiovascular disease), somatic comorbidity score, diabetes mellitus, and ADL scores was reduced to varying degrees (Supplementary Fig. S6). Hence, multiple sensitivity analyses for age, race and MMSE scores were performed by removing each study, but there was no change. Furthermore, we carried out meta-regression concerning items of age, sex, geographic region, sample size, NOS scores and follow-up time but failed to explain the source of heterogeneity. For the sensitivity analysis to test the robustness of the overall outcome, there appeared to be no significant difference in the results with any study removed except for depression and cancer (Supplementary Fig. S7).

In the secondary outcome, subgroup analysis based on age and NOS scores led to reduced heterogeneity for MMSE scores (Supplementary Fig. S6), and the outcome of the combination was stable in the sensitivity analysis (Supplementary Fig. S8).

### Assessment of publication bias

For the primary analysis, no influences of publication bias on the combined results were identified and the specific items are demonstrated in the Supplementary Materials. Whereas for MMSE scores (*P* = 0.040), there exists latent publication bias. Hence, the further trim-and-fill method was used and showed the authenticity and stability of the result (unchanged adjusted RR 0.934, 95% CI 0.917–0.950 for MMSE scores).

For the secondary outcome, the multiple sensitivity analysis exhibited no difference by eliminating any single study (Supplementary Materials). However, for MMSE scores, a bias was observed (*P* = 0.001). After the application of the trim-and-fill method, the combined estimate did not change (adjusted RR 0.930, 95% CI 0.915–0.946), which meant that the impact of publication bias was acceptable.

## Discussion

**Fig. 3:**
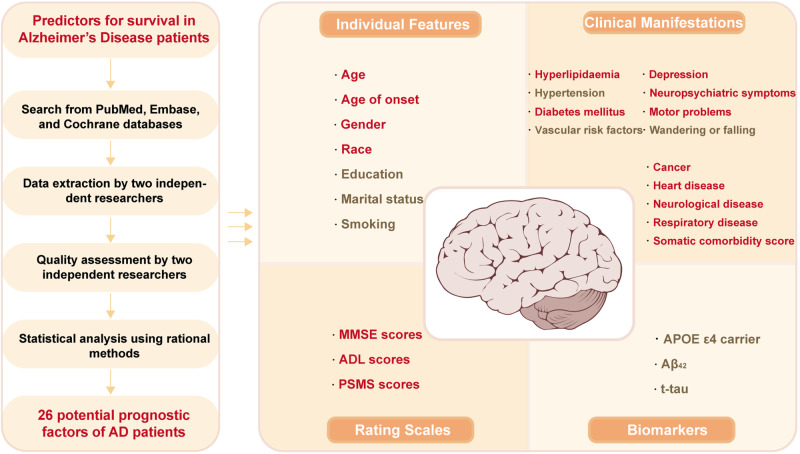
Workflow and main findings of the meta-analysis. AD Alzheimer’s disease, MMSE The Mini Mental State Examination, ADL Activity of Daily Living, PSMS Physical Self-Maintenance Scale, APOE Apolipoprotein E, CSF cerebrospinal fluid, Aβ β-amyloid, t-tau total tau protein.

To the best of our knowledge, there has been no meta-analysis summarizing prognostic factors for predicting the survival of AD patients from multiple dimensions. In this study, predictors, including demographic features, clinical features or comorbidities, rating scales and biomarkers, were investigated. In total, 26 probable prognostic factors were finally explored for AD survival, and 17 factors were identified as possibly related to the survival of AD (Fig. 3). Among them, hyperlipidaemia and higher MMSE scores were predictors of longer survival. However, males, features of movement disorders (including EPS) and cancer showed a worse prognosis. Moreover, older age, white race, a history of NPS, depression, heart disease, cerebrovascular disease, respiratory disease, higher somatic comorbidity score, diabetes mellitus, higher ADL scores and PSMS scores in patients also impaired AD survival. However, our results did not support the involvement of education, marital status, hypertension, wandering or falling, VRF, APOE genotype, Aβ_42_ or t-tau in AD survival. In the secondary analysis, we found that only movement disorders (including EPS), NPS and lower MMSE scores played a meaningful role in the deterioration of progress in AD patients, which was in accordance with our primary analysis. For intervenable symptoms such as NPS and diabetes mellitus, patients may benefit from regular treatments.

Less is known about the clinical value of various factors in AD progression or survival in the past, and many studies have attempted to spell out their associations [7–11, 86–92]. Compared with the results of former studies, either accordance or difference was observed in our analysis.

Our findings indicated that hyperlipidaemia was related to longer survival of AD, and yet other VRF, including overall VRF and some separate diseases such as smoking and hypertension, did not show a similar significant association. Earlier research stated that there was no difference in the rate of deterioration between people with and without VRF and assumed that VRF may contribute to the expression of AD initially but was not part of the underlying etiologic process [93, 94]. What amazed us was the negative link between hyperlipidaemia and mortality. It should be noted that elevations in blood lipids prolonged the survival of AD patients compared with those without hyperlipidaemia. Hyperlipidaemia has been identified as a risk factor for developing dementia. However, our analysis, which aggregated previous research findings, yielded conflicting results. These findings underscore the complexity of the role of hyperlipidaemia in the occurrence and progression of AD.

Other diseases, such as heart disease and cerebrovascular disease, which have been identified as driving forces in the process of dementia progression, were significantly associated with AD survival. Diabetes mellitus, one of the most prevalent comorbidities, plays an expediting role in disease progression [92, 95, 96], which might originate from an identical source as AD. This is not difficult to accept, as some researchers refer to AD as “diabetes of the brain” or “type-3 diabetes” [97]. In addition, movement disorders, often accompanied by functional defects, were discovered as a strong predictor of mortality and were associated with adverse outcomes [10, 98–100]. In our work, EPS influenced not only the survival of AD patients but also the quality of life since diagnosis, which is not hard to interpret because EPS, such as rigidity, tremor, and postural instability, means a loss of self-care to a certain extent. Similar to most of the following factors, cancer also significantly increased the risk of death in AD. One hypothesis was that the poor prognosis of patients with cancer shortened life expectancy, let alone those before the onset of AD. In terms of other clinical features, behavioral and psychological symptoms are common nonmotor symptoms during the natural history of AD, leading to distress for patients and their caregivers [101]. NPS and behavioral problems were proven to be detrimental to survival [10, 102], and similar results were found for depression, as in our work. In fact, how these manifestations interact remains unknown, and there is still controversy in some studies that disagree with the findings [93].

For demographic features, some items emerged as significant predictors of mortality. Older age and white race increased the risk of death in our study, which was in accordance with previous studies [7, 103, 104]. Meanwhile, a Framingham study suggested that due to “survival bias”, men appeared to have a lower risk for dementia, in which the included male participants who survived to 65 years old possessed a better physical condition [105]. The fact remains that once diagnosed with AD or other dementias, having male sex resulted in a worse prognosis compared to having female sex. Similarly, previous studies and our findings showed that a higher education level was not associated with decreased survival in AD [87, 106], in contrast to the evidence that a lower level of education was a risk factor for dementia [107]. The role of marital status should not be ignored, although we did not obtain a meaningful outcome because a former study reported that younger patients living alone exhibited a nearly threefold risk of death than those living with a family [108]. One explanation was that patients living alone were likely to be diagnosed at a later time than those who lived together with a spouse, which can influence the intervention measures to be taken.

Additionally, we found that cognitive decline and deterioration of personal self-care ability (such as ADL and PSMS scores) were associated with mortality risk in individuals with AD. Previous studies drew the same conclusion as well [109–112]. A result from a real-world cohort indicated that poorer baseline cognitive ability and short-term decline in functional ability independently predicted the transition from mild to more severe AD dementia [110]. Additionally, Aβ42 and tau are generally recognized as diagnostic biomarkers, but few studies have examined whether AD biomarkers are associated with mortality. In our analysis, no difference was observed for APOE ε4 carriers and different levels of CSF biomarkers in disease progression, similar to previous studies [113–115]. The possible reason was that growing evidence of shared molecular mechanisms between AD and atherosclerosis, showed an association with more cardiovascular mortality [34]. Notably, it was also reported that AD patients with extreme levels of CSF biomarkers exhibited worse clinical outcomes over time [114], which might be explained by more advanced disease that contributed to the risk of death. In addition, baseline plasma neurofilament light (NFL) chain was regarded as a predictor of cognitive decline, along with plasma tau in the late mild cognitive impairment (MCI) population [116].

The primary strength of our meta-analysis lies in its comprehensive and large-scale summary of prognostic factors for predicting survival in patients with AD from four dimensions. Another strength is that more high-quality prospective studies with nearly 300,000 AD patients were included, and stricter inclusion and exclusion criteria were used, which exhibited substantial power in drawing a conclusion. Moreover, we chose the most adjusted variables to decrease the impact of confounding factors that might influence the outcome. Last, a single type of dementia (Alzheimer’s disease) rather than multiple types of dementia was focused on to understand the course of the disease pertinently [9, 92, 113, 117]. Although some predictors that affected AD survival were identified, these results should be considered with caution due to several limitations. First, we excluded studies that reported different classifications of categorical data or reported ORs as estimate variables to avoid bias. Second, heterogeneity still existed in the analysis of age, race and MMSE scores after the application of multifarious methods, and the generated estimates of clinical type, depression and cancer were not robust in the sensitivity analysis on account of the restricted number of studies. Third, publication bias could not be ignored in that some studies only reported significant results, and the personal characteristics, follow-up time, and sample size varied among studies, although efforts have been made to take that into account. Fourth, the potential relationship between hyperlipidaemia and AD survival could not be interpreted clearly, indicating a need for more research on this topic. Finally, we did not discuss the influence of therapeutic measures on survival in AD patients since observational studies were the major study type within the scope of the search strategy and randomized control studies were incomplete. Similarly, genetic factors were not taken into consideration due to the complicated pathogenesis.

This meta-analysis comprehensively identified intervenable and unmodifiable risk factors for predicting survival in patients with AD from the dimensions of demographic features, clinical features or comorbidities, rating scales and biomarkers.

### Supplementary information

Supplementary materials

## Data Availability

All data analyzed during this study are included in the Supplementary Materials.

## References

[1] Kumar A, Singh A, Ekavali (2015). A review on Alzheimer’s disease pathophysiology and its management: an update. Pharm Rep.

[2] Du W, Tan J, Xu W, Chen J, Wang L (2016). Association between clusterin gene polymorphism rs11136000 and late-onset Alzheimer’s disease susceptibility: A review and meta-analysis of case-control studies. Exp Ther Med.

[3] Scheltens P, De Strooper B, Kivipelto M, Holstege H, Chételat G, Teunissen CE (2021). Alzheimer’s disease. Lancet.

[4] GBD 2019 Ageing Collaborators. (2022). Global, regional, and national burden of diseases and injuries for adults 70 years and older: systematic analysis for the Global Burden of Disease 2019 Study. BMJ.

[5] GBD 2019 Diseases and Injuries Collaborators. (2020). Global burden of 369 diseases and injuries in 204 countries and territories, 1990-2019: a systematic analysis for the Global Burden of Disease Study 2019. Lancet.

[6] Cummings J, Lee G, Nahed P, Kambar M, Zhong K, Fonseca J (2022). Alzheimer’s disease drug development pipeline: 2022. Alzheimers Dement.

[7] Brookmeyer R, Corrada MM, Curriero FC, Kawas C (2002). Survival following a diagnosis of Alzheimer disease. Arch Neurol.

[8] Jagger C, Clarke M, Stone A (1995). Predictors of survival with Alzheimer’s disease: a community-based study. Psychol Med.

[9] Brodaty H, Seeher K, Gibson L (2012). Dementia time to death: a systematic literature review on survival time and years of life lost in people with dementia. Int Psychogeriatr.

[10] Stern Y, Mayeux R, Sano M, Hauser WA, Bush T (1987). Predictors of disease course in patients with probable Alzheimer’s disease. Neurology.

[11] Rountree SD, Chan W, Pavlik VN, Darby EJ, Doody RS (2012). Factors that influence survival in a probable Alzheimer disease cohort. Alzheimers Res Ther.

[12] Sousa OV, Amaral TF (2017). Nutritional and functional status in survival of free-living mild Alzheimer’s disease patients. J Cachexia Sarcopenia Muscle.

[13] Zhou J, Yu JT, Wang HF, Meng XF, Tan CC, Wang J (2015). Association between stroke and Alzheimer’s disease: systematic review and meta-analysis. J Alzheimers Dis.

[14] Vázquez Justes D, Dakterzada F, Romero L, Ruiz-Julián M, Paul Arias M, Baraldés Rovira M (2021). Microbleeds and the risk of stroke and mortality in patients with alzheimer disease after 3 years of follow-up. Eur Stroke J.

[15] Ousset PJ, Nourhashemi F, Reynish E, Vellas B (2008). Nutritional status is associated with disease progression in very mild Alzheimer disease. Alzheimer Dis Associated Disord.

[16] Ferrari-Souza JP, Brum WS, Hauschild LA, da Ros LU, Lukasewicz Ferreira PC, Bellaver B (2024). Vascular risk burden is a key player in the early progression of Alzheimer’s disease. Neurobiol Aging.

[17] Rahmani J, Roudsari AH, Bawadi H, Clark C, Ryan PM, Salehisahlabadi A (2022). Body mass index and risk of Parkinson, Alzheimer, Dementia, and Dementia mortality: a systematic review and dose–response meta-analysis of cohort studies among 5 million participants. Nutritional Neurosci.

[18] Grønning H, Rahmani A, Gyllenborg J, Dessau R, Høgh P (2012). Does Alzheimer’s disease with early-onset progress faster than with late-onset? A case-control study of clinical progression and cerebrospinal fluid biomarkers. Eur J Neurol.

[19] Page MJ, McKenzie JE, Bossuyt PM, Boutron I, Hoffmann TC, Mulrow CD (2021). The PRISMA 2020 statement: an updated guideline for reporting systematic reviews. BMJ.

[20] Orsini N (2010). From floated to conventional confidence intervals for the relative risks based on published dose-response data. Comput Methods Prog Biomed.

[21] Hartung J, Knapp G (2001). On tests of the overall treatment effect in meta-analysis with normally distributed responses. Stat Med.

[22] Hartung J, Knapp G (2001). A refined method for the meta-analysis of controlled clinical trials with binary outcome. Stat Med.

[23] Burns A, Lewis G, Jacoby R, Levy R (1991). Factors affecting survival in Alzheimer’s disease. Psychological Med.

[24] Heyman A, Peterson B, Fillenbaum G, Pieper C (1996). The consortium to establish a registry for Alzheimer’s disease (CERAD). Part XIV: Demographic and clinical predictors of survival in patients with Alzheimer’s disease. Neurology.

[25] Stern Y, Brandt J, Albert M, Jacobs DM, Liu X, Bell K (1997). The absence of an apolipoprotein epsilon4 allele is associated with a more aggressive form of Alzheimer’s disease. Ann Neurol.

[26] Claus JJ, van Gool WA, Teunisse S, Walstra GJ, Kwa VI, Hijdra A (1998). Predicting survival in patients with early Alzheimer’s disease. Dement Geriatr Cogn Disord.

[27] White H, Pieper C, Schmader K (1998). The association of weight change in Alzheimer’s disease with severity of disease and mortality: a longitudinal analysis. J Am Geriatr Soc.

[28] McCann JJ, Hebert LE, Li Y, Wolinsky FD, Gilley DW, Aggarwal NT (2005). The effect of adult day care services on time to nursing home placement in older adults with Alzheimer’s disease. Gerontologist.

[29] Mehta KM, Yaffe K, Pérez-Stable EJ, Stewart A, Barnes D, Kurland BF (2008). Race/ethnic differences in AD survival in US Alzheimer’s Disease Centers. Neurology.

[30] Henneman WJ, Sluimer JD, Cordonnier C, Baak MM, Scheltens P, Barkhof F (2009). MRI biomarkers of vascular damage and atrophy predicting mortality in a memory clinic population. Stroke.

[31] Wattmo C, Wallin AK, Londos E, Minthon L (2011). Risk factors for nursing home placement in Alzheimer’s disease: a longitudinal study of cognition, ADL, service utilization, and cholinesterase inhibitor treatment. Gerontologist.

[32] Lopez OL, Becker JT, Chang YF, Sweet RA, Aizenstein H, Snitz B (2013). The long-term effects of conventional and atypical antipsychotics in patients with probable Alzheimer’s disease. Am J Psychiatry.

[33] Rabins PV, Schwartz S, Black BS, Corcoran C, Fauth E, Mielke M (2013). Predictors of progression to severe Alzheimer’s disease in an incidence sample. Alzheimers Dement.

[34] Boumenir A, Cognat E, Sabia S, Hourregue C, Lilamand M, Dugravot A (2019). CSF level of β-amyloid peptide predicts mortality in Alzheimer’s disease. Alzheimers Res Ther.

[35] Walsh JS, Welch HG, Larson EB (1990). Survival of outpatients with Alzheimer-type dementia. Ann Intern Med.

[36] Chui HC, Lyness SA, Sobel E, Schneider LS (1994). Extrapyramidal signs and psychiatric symptoms predict faster cognitive decline in Alzheimer’s disease. Arch Neurol.

[37] Stern Y, Albert M, Brandt J, Jacobs DM, Tang MX, Marder K (1994). Utility of extrapyramidal signs and psychosis as predictors of cognitive and functional decline, nursing home admission, and death in Alzheimer’s disease: Prospective analyses from the predictors study. Neurology.

[38] Stern Y, Tang MX, Denaro J, Mayeux R (1995). Increased risk of mortality in Alzheimer’s disease patients with more advanced educational and occupational attainment. Ann Neurol.

[39] Bowen JD, Malter AD, Sheppard L, Kukull WA, McCormick WC, Teri L (1996). Predictors of mortality in patients diagnosed with probable Alzheimer’s disease. Neurology.

[40] Geerlings MI, Deeg DJH, Schmand B, Lindeboom J, Jonker C (1997). Increased risk of mortality in Alzheimer’s disease patients with higher education? A replication study. Neurology.

[41] Stern Y, Tang MX, Albert MS, Brandt J, Jacobs DM, Bell K (1997). Predicting time to nursing home care and death in individuals with Alzheimer disease. J Am Med Assoc.

[42] Tilvis RS, Strandberg TE, Juva K (1998). Apolipoprotein E phenotypes, dementia and mortality in a prospective population sample. J Am Geriatr Soc.

[43] Claus JJ, Walstra GJM, Hijdra A, Van Royen EA, Verbeeten B, Van Gool WA (1999). Measurement of temporal regional cerebral perfusion with single-photon emission tomography predicts rate of decline in language function and survival in early Alzheimer’s disease. Eur J Nucl Med.

[44] Larson EB, Shadlen MF, Wang L, McCormick WC, Bowen JD, Teri L (2004). Survival after Initial Diagnosis of Alzheimer Disease. Ann Intern Med.

[45] Scarmeas N, Albert M, Brandt J, Blacker D, Hadjigeorgiou G, Papadimitriou A (2005). Motor signs predict poor outcomes in Alzheimer disease. Neurology.

[46] Scarmeas N, Brandt J, Albert M, Hadjigeorgiou G, Papadimitriou A, Dubois B (2005). Delusions and hallucinations are associated with worse outcome in Alzheimer disease. Arch Neurol.

[47] Suh GH, Yeon BK, Shah A, Lee JY (2005). Mortality in Alzheimer’s disease: A comparative prospective Korean study in the community and nursing homes. Int J Geriatr Psychiatry.

[48] Waring SC, Doody RS, Pavlik VN, Massman PJ, Chan W (2005). Survival among patients with dementia from a large multi-ethnic population. Alzheimer Dis Assoc Disord.

[49] Carcaillon L, Pérès K, Péré JJ, Helmer C, Orgogozo JM, Dartigues JF (2007). Fast cognitive decline at the time of dementia diagnosis: A major prognostic factor for survival in the community. Dement Geriatr Cogn Disord.

[50] Scarmeas N, Brandt J, Blacker D, Albert M, Hadjigeorgiou G, Dubois B (2007). Disruptive behavior as a predictor in Alzheimer disease. Arch Neurol.

[51] Bruandet A, Richard F, Bombois S, Maurage CA, Masse I, Amouyel P (2008). Cognitive decline and survival in Alzheimer’s disease according to education level. Dement Geriatr Cogn Disord.

[52] Helzner EP, Scarmeas N, Cosentino S, Tang MX, Schupf N, Stern Y (2008). Survival in Alzheimer disease: a multiethnic, population-based study of incident cases. Neurology.

[53] Hatoum HT, Thomas SK, Lin SJ, Lane R, Bullock R (2009). Predicting time to nursing home placement based on activities of daily living scores-a modelling analysis using data on Alzheimer’s disease patients receiving rivastigmine or donepezil. J Med Econ.

[54] Pavlik VN, Doody RS, Rountree SD, Darby EJ (2009). Vitamin E Use Is Associated with Improved Survival in an Alzheimer’s Disease Cohort. Dement Geriatr Cogn Disord.

[55] Zhou B, Zhao Q, Teramukai S, Ding D, Guo Q, Fukushima M (2010). Executive function predicts survival in Alzheimer disease: A study in Shanghai. J Alzheimers Dis.

[56] Musicco M, Palmer K, Russo A, Caltagirone C, Adorni F, Pettenati C (2011). Association between prescription of conventional or atypical antipsychotic drugs and mortality in older persons with Alzheimer’s disease. Dement Geriatr Cogn Disord.

[57] Go SM, Lee KS, Seo SW, Chin J, Kang SJ, Moon SY (2013). Survival of alzheimer’s disease patients in Korea. Dement Geriatr Cogn Disord.

[58] Degerman Gunnarsson M, Lannfelt L, Ingelsson M, Basun H, Kilander L (2014). High tau levels in cerebrospinal fluid predict rapid decline and increased dementia mortality in Alzheimer’s disease. Dement Geriatr Cogn Disord.

[59] Nägga K, Wattmo C, Zhang Y, Wahlund LO, Palmqvist S (2014). Cerebral inflammation is an underlying mechanism of early death in Alzheimer’s disease: A 13-year cause-specific multivariate mortality study. Alzheimers Res Ther.

[60] Wattmo C, Londos E, Minthon L (2014). Risk factors that affect life expectancy in Alzheimer’s disease: a 15-year follow-up. Dement Geriatr Cogn Disord.

[61] Benedictus MR, Prins ND, Goos JDC, Scheltens P, Barkhof F, Van Der Flier WM (2015). Microbleeds, Mortality, and Stroke in Alzheimer Disease The MISTRAL Study. JAMA Neurol.

[62] Lin FC, Chuang YS, Hsieh HM, Lee TC, Chiu KF, Liu CK (2015). Early statin use and the progression of Alzheimer disease: A total population-based case-control study. Medicine.

[63] Wattmo C, Londos E, Minthon L (2015). Longitudinal associations between survival in Alzheimer’s disease and cholinesterase inhibitor use, progression, and community-based services. Dement Geriatr Cogn Disord.

[64] Degerman Gunnarsson M, Ingelsson M, Blennow K, Basun H, Lannfelt L, Kilander L (2016). High tau levels in cerebrospinal fluid predict nursing home placement and rapid progression in Alzheimer’s disease. Alzheimers Res Ther.

[65] Nielsen RE, Lolk A, Valentin JB, Andersen K (2016). Cumulative dosages of antipsychotic drugs are associated with increased mortality rate in patients with Alzheimer’s dementia. Acta Psychiatr Scandinavica.

[66] Mueller C, Huntley J, Stubbs B, Sommerlad A, Carvalho AF, Perera G (2017). Associations of Neuropsychiatric Symptoms and Antidepressant Prescription with Survival in Alzheimer’s Disease. J Am Med Dir Assoc.

[67] Black CM, Fillit H, Xie L, Hu X, Kariburyo MF, Ambegaonkar BM (2018). Economic Burden, Mortality, and Institutionalization in Patients Newly Diagnosed with Alzheimer’s Disease. J Alzheimers Dis.

[68] Chu CS, Li WR, Huang KL, Su PY, Lin CH, Lan TH (2018). The use of antipsychotics is associated with lower mortality in patients with Alzheimer’s disease: A nationwide population-based nested case-control study in Taiwan. J Psychopharmacol.

[69] Giil LM, Aarsland D, Hellton K, Lund A, Heidecke H, Schulze-Forster K (2018). Antibodies to multiple receptors are associated with neuropsychiatric symptoms and mortality in Alzheimer’s disease: A longitudinal study. J Alzheimers Dis.

[70] Ku LJE, Li CY, Sun Y (2018). Can Persistence With Cholinesterase Inhibitor Treatment Lower Mortality and Health-Care Costs Among Patients With Alzheimer’s Disease? A Population-Based Study in Taiwan. Am J Alzheimers Dis other Dement.

[71] Mueller C, Perera G, Hayes RD, Shetty H, Stewart R (2018). Associations of acetylcholinesterase inhibitor treatment with reduced mortality in Alzheimer’s disease: A retrospective survival analysis. Age Ageing.

[72] Nielsen RE, Valentin JB, Lolk A, Andersen K (2018). Effects of antipsychotics on secular mortality trends in patients with Alzheimer’s disease. J Clin Psychiatry.

[73] Rhodius-Meester HFM, Liedes H, Koene T, Lemstra AW, Teunissen CE, Barkhof F (2018). Disease-related determinants are associated with mortality in dementia due to Alzheimer’s disease. Alzheimers Res Ther.

[74] Chen TB, Weng SC, Chou YY, Lee YS, Liang CK, Lin CS (2019). Predictors of Mortality in the Oldest Old Patients with Newly Diagnosed Alzheimer Disease in a Residential Aged Care Facility. Dement Geriatr Cogn Disord.

[75] Linna M, Vuoti S, Silander K, Hörhammer I, Halminen O, Mikkola T (2019). Impact of Anti-Dementia Medication on the Risk of Death and Causes of Death in Alzheimer’s Disease. J Alzheimers Dis.

[76] Chen NC, Liang CK, Yin CH, Lin YT, Lee CC, Chen CL (2020). Effects of Socioeconomic Status on Alzheimer Disease Mortality in Taiwan. Am J Geriatr Psychiatry.

[77] de Sousa OV, Mendes J, Amaral TF (2020). Nutritional and Functional Indicators and Their Association With Mortality Among Older Adults With Alzheimer’s Disease. Am J Alzheimers Dis other Dement.

[78] Zhang YQ, Wang CF, Xu G, Zhao QH, Xie XY, Cui HL (2020). Mortality of Alzheimer’s Disease Patients: A 10-Year Follow-up Pilot Study in Shanghai. Can J Neurological Sci.

[79] Gottesman RT, Kociolek A, Fernandez K, Cosentino S, Devanand DP, Stern Y (2021). Association between Early Psychotic Symptoms and Alzheimer’s Disease Prognosis in a Community-Based Cohort. J Alzheimers Dis.

[80] Liew TM (2021). Neuropsychiatric symptoms in early stage of Alzheimer’s and non-Alzheimer’s dementia, and the risk of progression to severe dementia. Age Ageing.

[81] Rajamaki B, Hartikainen S, Tolppanen AM (2021). The effect of comorbidities on survival in persons with Alzheimer’s disease: a matched cohort study. BMC Geriatrics.

[82] Wattmo C, Blennow K, Hansson O (2021). Cerebrospinal Fluid Biomarker Levels as Markers for Nursing Home Placement and Survival Time in Alzheimer’s Disease. Curr Alzheimer Res.

[83] Armstrong MJ, Song S, Kurasz AM, Li Z (2022). Predictors of Mortality in Individuals with Dementia in the National Alzheimer’s Coordinating Center. J Alzheimers Dis.

[84] Ono R, Uchida K, Nakatsuka K, Megumi M, Fukuda H (2022). Economic Status and Mortality in Patients with Alzheimer’s Disease in Japan: The Longevity Improvement and Fair Evidence Study. J Am Med Dir Assoc.

[85] van Loenhoud AC, Groot C, Bocancea DI, Barkhof F, Teunissen C, Scheltens P (2022). Association of Education and Intracranial Volume With Cognitive Trajectories and Mortality Rates Across the Alzheimer Disease Continuum. Neurology.

[86] Felbecker A, Tettenborn B (2016). Influence of Vascular Risk Factors on Cause and Progression of Alzheimer’s Disease. Aktuelle Neurologie.

[87] Di Santo S, Musicco M (2011). Lifestyle and rate of progression of cognitive decline: Results of the SINDEM cohort study. J Alzheimer’s Dis.

[88] Todd S, Barr S, Roberts M, Passmore AP (2013). Survival in dementia and predictors of mortality: a review. Int J Geriatr Psychiatry.

[89] Lee JH (2015). Clinical factors influencing the rate of progression of dementia: A retrospective review from 50 geriatric hospitals in korea. Alzheimers Dement.

[90] Sathe AB, Koh M, Wu HY, Leong I, Ali NB, Chin JJ (2015). Predictors of survival in moderate to advanced dementia: A retrospective review. Ann Acad Med Singap.

[91] Alonso A, Jacobs DR, Menotti A, Nissinen A, Dontas A, Kafatos A (2009). Cardiovascular risk factors and dementia mortality: 40 years of follow-up in the Seven Countries Study. J Neurol Sci.

[92] van de Vorst IE, Koek HL, de Vries R, Bots ML, Reitsma JB, Vaartjes I (2016). Effect of Vascular Risk Factors and Diseases on Mortality in Individuals with Dementia: A Systematic Review and Meta-Analysis. J Am Geriatr Soc.

[93] Drachman DA, O’Donnell BF, Lew RA, Swearer JM (1990). The prognosis in Alzheimer’s disease. ‘How far’ rather than ‘how fast’ best predicts the course. Arch Neurol.

[94] Kalaria RN, Ballard C (1999). Overlap between pathology of Alzheimer disease and vascular dementia. Alzheimer Dis Assoc Disord.

[95] Wang G (2019). Mortality Of Alzheimer’s Disease Patients In Shanghai: Findings From A Clinic-Based Ten-Year Follow-Up Study. Alzheimers Dement.

[96] Norton S, Matthews FE, Barnes DE, Yaffe K, Brayne C (2014). Potential for primary prevention of Alzheimer’s disease: an analysis of population-based data. Lancet Neurol.

[97] Nguyen TT, Ta QTH, Nguyen TKO, Nguyen TTD, Giau VV (2020). Type 3 Diabetes and Its Role Implications in Alzheimer’s Disease. Int J Mol Sci.

[98] Al-Harrasi AM, Iqbal E, Tsamakis K, Lasek J, Gadelrab R, Soysal P (2021). Motor signs in Alzheimer’s disease and vascular dementia: Detection through natural language processing, co-morbid features and relationship to adverse outcomes. Exp Gerontol.

[99] Samson WN, van Duijn CM, Hop WC, Hofman A (1996). Clinical features and mortality in patients with early-onset Alzheimer’s disease. Eur Neurol.

[100] Lopez OL, Wisnieski SR, Becker JT, Boller F, DeKosky ST (1997). Extrapyramidal signs in patients with probable Alzheimer disease. Arch Neurol.

[101] Ballard C, Day S, Sharp S, Wing G, Sorensen S (2008). Neuropsychiatric symptoms in dementia: importance and treatment considerations. Int Rev Psychiatry.

[102] Herrmann N, Harimoto T, Balshaw R, Lanctôt KL, Bacher Y, Bailey P (2015). Risk factors for progression of Alzheimer disease in a Canadian population: The Canadian Outcomes Study in Dementia (COSID). Can J Psychiatry.

[103] Rountree S, Chan W, Pavlik V, Darby E, Doody R (2011). Factors that influence survival in alzheimer’s patients. Alzheimer’s Dement.

[104] Barr S, Passmore AP, Todd S (2010). Excess mortality associated with Alzheimer’s disease in a community-dwelling population attending a Memory Clinic. Eur Geriatr Med.

[105] Seshadri S, Wolf PA, Beiser A, Au R, McNulty K, White R (1997). Lifetime risk of dementia and Alzheimer’s disease. The impact of mortality on risk estimates in the Framingham Study. Neurology.

[106] Paradise M, Cooper C, Livingston G (2009). Systematic review of the effect of education on survival in Alzheimer’s disease. Int Psychogeriatr.

[107] Yu JT, Xu W, Tan CC, Andrieu S, Suckling J, Evangelou E (2020). Evidence-based prevention of Alzheimer’s disease: systematic review and meta-analysis of 243 observational prospective studies and 153 randomised controlled trials. J Neurol Neurosurg Psychiatry.

[108] Wattmo C, Londos E (2018). Predictors Of Mortality In Early- Versus Late-Onset Alzheimer’s Disease: An 18-Year Follow-Up. Alzheimers Dement.

[109] Soto ME, Andrieu S, Cantet C, Reynish E, Ousset PJ, Arbus C (2008). Predictive value of rapid decline in mini mental state examination in clinical practice for prognosis in Alzheimer’s disease. Dement Geriatr Cogn Disord.

[110] Reed C, Lebrec J, Andrews JS, Bruno G, Jones RW (2016). Predictors of Disease Progression in Mild Alzheimer’s Disease Dementia Patients – Results From the Geras Real World Cohort and Expedition Trials. Value Health.

[111] Wilson RS, Li Y, Aggarwal NT, McCann JJ, Gilley DW, Bienias JL (2006). Cognitive decline and survival in Alzheimer’s disease. Int J Geriatr Psychiatry.

[112] Wattmo C, Londos E, Minthon L (2015). Various measures of progression predicting mortality in Alzheimer’s disease. Alzheimers Dement.

[113] Allan CL, Ebmeier KP (2011). The influence of ApoE4 on clinical progression of dementia: A meta-analysis. Int J Geriatr Psychiatry.

[114] Wallin A, Blennow K, Zetterberg H, Londos E, Minthon L, Hansson O (2010). CSF biomarkers predict a more malignant outcome in Alzheimer’s disease. Eur J Neurol.

[115] Liu E, Ashaye A, Travers K, Strand L, Tanna GL, Wyman BT (2014). A prospective, systematic literature review and meta-analyses to evaluate brain amyloid by positron emission tomography(PET) imaging as a biomarker of Alzheimer’s disease (AD) progression. Alzheimers Dement.

[116] Soares H, Feng S, Florian H, Gold M, Lon HK, Mendonca N (2018). Plasma Tau And Neurofilament Light Chain As A Prognostic Biomarker Of Disease Progression In Early Alzheimer’s Disease. Alzheimers Dement.

[117] Cepoiu-Martin M, Tam-Tham H, Patten S, Maxwell CJ, Hogan DB (2016). Predictors of long-term care placement in persons with dementia: a systematic review and meta-analysis. Int J Geriatr Psychiatry.

