# Supplementary material for: Predictors for survival in patients with Alzheimer’s disease: a large comprehensive meta-analysis
Source: Transl Psychiatry. 2024 Apr 10;14:184. doi: 10.1038/s41398-024-02897-w (PMC11006915; doi:10.1038/s41398-024-02897-w)
Supplement: Supplementary file 1 — Supplementary materials [file 41398_2024_2897_MOESM1_ESM.docx]

Predictors for Survival in Patients with Alzheimer’s Disease: A Large Comprehensive Meta-analysis

Xiaoting Zheng^1^, Shichan Wang^1^, Jingxuan Huang^1^, Chunyu Li^1,*^, Huifang Shang^1,*^

^1^Department of Neurology, Laboratory of Neurodegenerative Disorders, National Clinical Research Center for Geriatrics, West China Hospital, Sichuan University, Chengdu, 610041, China.

^*^Corresponding author:

Huifang Shang, Postal address: No.37, Guoxue Lane, 610041, Chengdu, Sichuan, China. E-mail: [hfshang2002@126.com](mailto:hfshang2002@126.com). Telephone Number: 0086-18980602127

Chunyu Li, Postal address: No.37, Guoxue Lane, 610041, Chengdu, Sichuan, China. E-mail: lichunyu.scu@qq.com. Telephone Number: 0086-18980600976

**Supplementary information**

[Supplementary statements 3](#_Toc148796542)

[Supplementary table 1. PRISMA structured checklist for systematic review and meta-analysis 4](#_Toc148796543)

[Supplementary table 2. Definition of several prognostic factors and endpoints in the meta-analysis 8](#_Toc148796544)

[Supplementary table 3. Characteristics of included studies in the meta-analysis 9](#_Toc148796545)

[Supplementary table 4. Quality assessment and confounding factors of included studies in the meta-analysis 17](#_Toc148796546)

[Supplementary table 5. Meta-analysis of potential factors for survival in patients with AD 20](#_Toc148796547)

[Supplementary figure 1. Forest plot of demographic features as prognostic factors in AD patients. 22](#_Toc148796548)

[Supplementary figure 2-A. Forest plot of clinical features or comorbidities as prognostic factors in AD patients. 23](#_Toc148796549)

[Supplementary figure 2-B. Forest plot of clinical features or comorbidities as prognostic factors in AD patients. 24](#_Toc148796550)

[Supplementary figure 3. Forest plot of rating scales as prognostic factors in AD patients. 25](#_Toc148796551)

[Supplementary figure 4. Forest plot of biomarkers as prognostic factors in AD patients. 26](#_Toc148796552)

[Supplementary figure 5. Forest plot for secondary outcome of prognostic factors in AD patients. 27](#_Toc148796553)

[Supplementary figure 6-A. Forest plot for subgroup analysis of prognostic factors in AD patients. 28](#_Toc148796554)

[Supplementary figure 6-B. Forest plot for subgroup analysis of prognostic factors in AD patients. 29](#_Toc148796555)

[Supplementary figure 7-A. Sensitive analysis of prognostic factors in AD patients. 30](#_Toc148796556)

[Supplementary figure 7-B. Sensitive analysis of prognostic factors in AD patients. 31](#_Toc148796557)

[Supplementary figure 7-C. Sensitive analysis of prognostic factors in AD patients. 32](#_Toc148796558)

[Supplementary figure 8. Sensitive analysis for secondary outcome of prognostic factors in AD patients. 33](#_Toc148796559)

[Reference 34](#_Toc148796560)

Supplementary statements

As supplementary materials to expound details of this work, here we listed included factors and results of publication bias test.

**Included potential factors**

The potential factors from four dimensions were shown as follows and in supplementary table 2: age (baseline age and age of onset), sex, race, education level, marital status (living alone or not), smoking (smoking or not), the Mini Mental State Examination (MMSE) scores, Activity of Daily Living (ADL) scores, Physical Self-Maintenance Scale (PSMS) scores, Apolipoprotein E (APOE) genotype, cerebrospinal fluid (CSF) β-amyloid (Aβ), total tau protein (t-tau), somatic comorbidity score, wandering or falling, movement disorders (including extrapyramidal signs, EPS), neuropsychiatric symptoms (NPS), depression, heart disease (including vascular and non-vascular disease), cerebrovascular disease, respiratory disease, hypertension, hyperlipidaemia, diabetes mellitus, cancer and vascular risk factors (VRF).

**Publication bias items**

In the primary analysis, publication bias of the following items were stated: baseline age (P=0.791), age of onset (P=0.814), sex (P=0.580), race (P=0.800), EPS (P=0.178), movement disorders (including EPS) (P=0.300), somatic comorbidity score (P=0.958), NPS (P=0.752), depression (P=0.752), heart disease (P=0.990), cerebrovascular disease (P=0.743), respiratory disease (P=0.895), hyperlipidaemia (P=1.00), diabetes mellitus (P=0.792), cancer (P=0.408), ADL scores (P=0.102) and PSMS scores (P=0.534).

In the secondary outcome, the Egger test indicated absence of publication bias among studies for movement disorders (including EPS) (P=0.642) and NPS (P=0.511).

Supplementary table 1. PRISMA [structured checklist](http://www.equator-network.org/reporting-guidelines/consort-2010-statement-updated-guidelines-for-reporting-parallel-group-randomised-trials/) for systematic review and meta-analysis

| **Section and Topic** | **Item #** | **Checklist item** | **Location where item is reported** |
| --- | --- | --- | --- |
| **TITLE** | | |  |
| Title | 1 | Identify the report as a systematic review. | 1 |
| **ABSTRACT** | | |  |
| Abstract | 2 | See the PRISMA 2020 for Abstracts checklist. | 2 |
| **INTRODUCTION** | | |  |
| Rationale | 3 | Describe the rationale for the review in the context of existing knowledge. | 3 |
| Objectives | 4 | Provide an explicit statement of the objective(s) or question(s) the review addresses. | 4 |
| **METHODS** | | |  |
| Eligibility criteria | 5 | Specify the inclusion and exclusion criteria for the review and how studies were grouped for the syntheses. | 4 |
| Information sources | 6 | Specify all databases, registers, websites, organisations, reference lists and other sources searched or consulted to identify studies. Specify the date when each source was last searched or consulted. | 4 |
| Search strategy | 7 | Present the full search strategies for all databases, registers and websites, including any filters and limits used. | 4 |
| Selection process | 8 | Specify the methods used to decide whether a study met the inclusion criteria of the review, including how many reviewers screened each record and each report retrieved, whether they worked independently, and if applicable, details of automation tools used in the process. | 4 |
| Data collection process | 9 | Specify the methods used to collect data from reports, including how many reviewers collected data from each report, whether they worked independently, any processes for obtaining or confirming data from study investigators, and if applicable, details of automation tools used in the process. | 5 |
| Data items | 10a | List and define all outcomes for which data were sought. Specify whether all results that were compatible with each outcome domain in each study were sought (e.g. for all measures, time points, analyses), and if not, the methods used to decide which results to collect. | 5 |
|  | 10b | List and define all other variables for which data were sought (e.g. participant and intervention characteristics, funding sources). Describe any assumptions made about any missing or unclear information. | 5 |
| Study risk of bias assessment | 11 | Specify the methods used to assess risk of bias in the included studies, including details of the tool(s) used, how many reviewers assessed each study and whether they worked independently, and if applicable, details of automation tools used in the process. | 5 |
| Effect measures | 12 | Specify for each outcome the effect measure(s) (e.g. risk ratio, mean difference) used in the synthesis or presentation of results. | 6 |
| Synthesis methods | 13a | Describe the processes used to decide which studies were eligible for each synthesis (e.g. tabulating the study intervention characteristics and comparing against the planned groups for each synthesis (item #5)). | 6 |
|  | 13b | Describe any methods required to prepare the data for presentation or synthesis, such as handling of missing summary statistics, or data conversions. | 6 |
|  | 13c | Describe any methods used to tabulate or visually display results of individual studies and syntheses. | 6 |
|  | 13d | Describe any methods used to synthesize results and provide a rationale for the choice(s). If meta-analysis was performed, describe the model(s), method(s) to identify the presence and extent of statistical heterogeneity, and software package(s) used. | 6 |
|  | 13e | Describe any methods used to explore possible causes of heterogeneity among study results (e.g. subgroup analysis, meta-regression). | 6 |
|  | 13f | Describe any sensitivity analyses conducted to assess robustness of the synthesized results. | 6 |
| Reporting bias assessment | 14 | Describe any methods used to assess risk of bias due to missing results in a synthesis (arising from reporting biases). | 6 |
| Certainty assessment | 15 | Describe any methods used to assess certainty (or confidence) in the body of evidence for an outcome. | 6 |
| **RESULTS** | | |  |
| Study selection | 16a | Describe the results of the search and selection process, from the number of records identified in the search to the number of studies included in the review, ideally using a flow diagram. | 7 |
|  | 16b | Cite studies that might appear to meet the inclusion criteria, but which were excluded, and explain why they were excluded. | 7 |
| Study characteristics | 17 | Cite each included study and present its characteristics. | 7 |
| Risk of bias in studies | 18 | Present assessments of risk of bias for each included study. | 7 |
| Results of individual studies | 19 | For all outcomes, present, for each study: (a) summary statistics for each group (where appropriate) and (b) an effect estimate and its precision (e.g. confidence/credible interval), ideally using structured tables or plots. | 7 |
| Results of syntheses | 20a | For each synthesis, briefly summarise the characteristics and risk of bias among contributing studies. | 7 |
|  | 20b | Present results of all statistical syntheses conducted. If meta-analysis was done, present for each the summary estimate and its precision (e.g. confidence/credible interval) and measures of statistical heterogeneity. If comparing groups, describe the direction of the effect. | 7 |
|  | 20c | Present results of all investigations of possible causes of heterogeneity among study results. | 9 |
|  | 20d | Present results of all sensitivity analyses conducted to assess the robustness of the synthesized results. | 9 |
| Reporting biases | 21 | Present assessments of risk of bias due to missing results (arising from reporting biases) for each synthesis assessed. | 10 |
| Certainty of evidence | 22 | Present assessments of certainty (or confidence) in the body of evidence for each outcome assessed. | 10 |
| **DISCUSSION** | | |  |
| Discussion | 23a | Provide a general interpretation of the results in the context of other evidence. | 10 |
|  | 23b | Discuss any limitations of the evidence included in the review. | 13 |
|  | 23c | Discuss any limitations of the review processes used. | 13 |
|  | 23d | Discuss implications of the results for practice, policy, and future research. | 13 |
| **OTHER INFORMATION** | | |  |
| Registration and protocol | 24a | Provide registration information for the review, including register name and registration number, or state that the review was not registered. | 4 |
|  | 24b | Indicate where the review protocol can be accessed, or state that a protocol was not prepared. | 4 |
|  | 24c | Describe and explain any amendments to information provided at registration or in the protocol. | 4 |
| Support | 25 | Describe sources of financial or non-financial support for the review, and the role of the funders or sponsors in the review. | 15 |
| Competing interests | 26 | Declare any competing interests of review authors. | 14 |
| Availability of data, code and other materials | 27 | Report which of the following are publicly available and where they can be found: template data collection forms; data extracted from included studies; data used for all analyses; analytic code; any other materials used in the review. | 15 |

Supplementary table 2. Definition of several prognostic factors and endpoints in the meta-analysis

| **Prognostic factors and endpoints** | **Interpretation** |
| --- | --- |
| movement disorders (including EPS) | any movement disorders including voice changes, facial immobility, resting tremor, rigidity, posture and gait abnormalities, and body bradykinesia-hypokinesia |
| NPS | including four types except depression: behavioural problem, specific hallucinations or delusions, psychosis, mood disorder, any of the above symptoms |
| cerebrovascular disease | any kind of cerebrovascular disease other than dementia |
| somatic comorbidity score | score based on cardiovascular disease, cancer, infection, diabetes, epilepsy, lower respiratory disease and other somatic diseases without investigating any one of which individually. |
| VRF | presence of one or more factors included history of diabetes mellitus, hypertension, myocardial infarction, transient ischemic attacks or stroke. |
| cognitive decline | a decline of ≥4 points/year in the MMSE score or a MMSE score of 15 as an end point. |

Abbreviations: EPS, extrapyramidal signs; NPS, neuropsychiatric symptoms; VRF, vascular risk factors.

Supplementary table 3. Characteristics of included studies in the meta-analysis

| Author | Year | Country | Case | Diagnosis criteria | Factors included | Endpoints | Mean/Median age (year) | sex (female, %) | Follow-up time (year) | Mean disease duration (year) | Median survival time (year) |
| --- | --- | --- | --- | --- | --- | --- | --- | --- | --- | --- | --- |
| Walsh *et al*(1) | 1990 | America | 126 | DSM-III | wandering or falling, NPS | death | 77.6^a^  73.9^b^ | 72 | at least 6 years | 3.8 | 5.3^a^  9.3^b^ |
| Burns *et al*(2) | 1991 | England | 178 | NINCDS-ADRDA, DSM-III-R, ICD 10 code for AD | age, gender, MMSE, depression | death | 80.4^a^  75.2^b^ | 79.2 | 1.53 | 5.29 | NK |
| Stern *et al*(3) | 1994 | America | 236 | NINCDS-ADRDA | movement disorders (including EPS), NPS | cognitive disability, functional disability, equivalent institutional care | 73.1^a^ | 59.3 | at least 0.5 year | 6.9 | NK |
| Chui *et al*(4) | 1994 | America | 135 | NINCDS-ADRDA | movement disorders (including EPS) | cognitive decline | 72.9^a^  69.0^b^ | 70 | 0.55 | 3.9 | NK |
| Stern *et al*(5) | 1995 | America | 246 | DSM-III-R, NINCDS-ADRDA | age, gender, education | death | 83.9^a^ | 75.2 | at least 1 year | NK | NK |
| Bowen *et al*(6) | 1996 | America | 327 | NINCDS-ADRDA | age, gender, smoking, weight loss, MMSE, wandering or falling, NPS, heart disease, hypertension, diabetes mellitus | death | 79.0^a^ | 64 | 3.3 | 2.6 | NK |
| Heyman *et al*(7) | 1996 | America | 1036 | NINCDS-ADRDA | gender, race, education, marital status, MMSE, ADL | death | 73.0^a^ | 58 | 3.2 | 3.8 | NK |
| Geerlings *et al*(8) | 1997 | The Netherlands | 66 | NINCDS-ADRDA | age, gender | death | 78.2^a^ for survived and 79.4^a^ for died | 80.3 | 4.0 | NK | NK |
| Stern *et al*(9) | 1997 | America | 236 | NINCDS-ADRDA | gender, MMSE, APOE ε4, movement disorders (including EPS), NPS | death | 73.1^a^ | 59.3 | at least 1 year | 3.9 | NK |
| Stern *et al*(10) | 1997 | America | 99 | NINCDS-ADRDA | APOE ε4 | death | 71.3^a^  67.3^b^ | 47.5 | 6.0 | 4.0 | NK |
| Tilvis *et al*(11) | 1998 | Finland | 41 | DSM-III-R | APOE ε4 | death | subgroup | NK | 5.0 | NK | NK |
| White *et al*(12) | 1998 | America | 633 | NINCDS-ADRDA | age, race, education, marital status, weight loss | death | 71.0^a^ for men and 72.0^a^ for women | 57 | 2.3 | NK | NK |
| Claus *et al*(13) | 1998 | The Netherlands | 163 | NINCDS-ADRDA | gender, ADL, movement disorders (including EPS), NPS | death | 79.2^a^ for probable AD and 78.9^a^ for possible AD | 59.5 | 4.3 | 2.2 for probable AD and 3.0 for possible AD | NK |
| Claus *et al*(14) | 1999 | The Netherlands | 69 | NINCDS-ADRDA | depression | death | 79.8^a^ | 52.2 | 0.5-5.5 | 3.16 | 3.52 |
| Larson *et al*(15) | 2004 | America | 521 | DSM-III-R, NINCDS-ADRDA | age of onset, movement disorders (including EPS), NPS, wandering or falling, depression, cerebrovascular disease, heart disease, hypertension, diabetes mellitus | death | subgroup | 65.5 | 5.2 | subgroup | 4.2 for men and 5.7 for women |
| Scarmeas *et al*(16) | 2005 | multiple countries | 533 | DSM-III-R, NINCDS-ADRDA | movement disorders (including EPS) | cognitive endpoint, institutionalization, death | 73.9^a^ | 61 | 3.05 | NK | 6.3 |
| Suh *et al*(17) | 2005 | Korea | 252 | DSM-IV, NINCDS-ADRDA | age, gender, education, MMSE, movement disorders (including EPS), VRF | death | 80.3^a^ | 82.1 | at least 1 year | 4.9 | NK |
| Waring *et al*(18) | 2005 | America | 640 | NINCDS-ADRDA | gender | death | 75.0^a^  72.0^b^ | NK | 5.7 | 3 | 5.7 |
| Scarmeas *et al*(19) | 2005 | multiple countries | 456 | DSM-III-R, NINCDS-ADRDA | NPS | cognitive outcome, functional outcome, institutionalization, death | 74.0^a^ | 59 | 4.5 | NK | NK |
| McCann *et al*(20) | 2005 | America | 416 | NINCDS-ADRDA | age, gender, MMSE | NHP | 81.5^a^ for day care and 79.3^a^ for no day care | 68.6 | 3.6 and 3.4 | NK | NK |
| Carcaillon *et al*(21) | 2007 | France | 245 | NINCDS-ADRDA | age, gender | death | 78.8^a^  85.5^b^ | 69.8 | 13.0 | NK | NK |
| Scarmeas *et al*(22) | 2007 | multiple countries | 497 | DSM-III-R, NINCDS-ADRDA | NPS | cognitive outcome, functional outcome, institutionalization, death | 73.8^a^ | 60 | 4.4 | 4.1 | NK |
| Bruandet *et al(23)* | 2008 | France | 670 | NINCDS-ADRDA | age, gender | death | subgroup | 64.5 | 3.0 | NK | NK |
| Helzner *et al*(24) | 2008 | America | 323 | DSM-III-R, NINCDS-ADRDA | gender, APOE ε4, hypertension, cerebrovascular disease, diabetes mellitus | death | 87.0^a^  83.0^b^ | 69.7 | 4.1 | NK | NK |
| Mehta *et al*(25) | 2008 | America | 30916 | NINCDS-ADRDA | race | death | 77.7^a^ | 65 | 2.4 | NK | 4.8 |
| Pavlik *et al*(26) | 2009 | America | 847 | NINCDS-ADRDA | age, gender, race | death | 73.5^a^ | 67.3 | 0.5-15.0 | 3.8 | 5.5 |
| Henneman *et al*(27) | 2009 | The Netherlands | 357 | NINCDS-ADRDA | cerebrovascular disease | death | 71.0^a^ for deceased and 66.0^a^ for alive | 45 | 2.6 | NK | NK |
| Hatoum *et al*(28) | 2009 | America | 800 | DSM-IV, NINCDS-ADRDA | age, gender, MMSE | NHP | 75.4^a^ | 69.7 | 2.0 | NK | NK |
| Zhou *et al*(29) | 2010 | China | 467 | DSM-IV, NINCDS-ADRDA | age, age of onset, gender, education, smoking, MMSE, wandering or falling, cerebrovascular disease, heart disease, hypertension, hyperlipidaemia, diabetes mellitus | death | 72.8^a^  71.2^b^ | 60.4 | 2.6 | NK | NK |
| Musicco *et al*(30) | 2011 | Italy | 4369 | NINCDS-ADRDA | age, gender, cerebrovascular disease, heart disease, cancer | death | 78.5^a^ | 65 | 2.0 | NK | NK |
| Wattmo *et al*(31) | 2011 | Sweden | 880 | DSM-IV, NINCDS-ADRDA | age, gender, MMSE, ADL, APOE ε4 | NHP | 76.4^a^, 72.9^b^ for NHP and 74.7^a^, 71.8^b^ for not-NHP | 63 | 4.7 | NK | 4.7 |
| Rountree *et al*(32) | 2012 | America | 641 | NINCDS-ADRDA | age, gender, ADL, PSMS | death | 73.0^a^ | 68 | 3.0 | 3.7 | 11.3 |
| Go *et al*(33) | 2013 | Korea | 724 | NINCDS-ADRDA | age of onset, gender, diabetes mellitus | death | 71.3^a^  68.5^b^ | 70.7 | 7.3^a^  10.2^b^ | NK | 5.7^a^  8.5^a^ |
| Lopez *et al*(34) | 2013 | America | 957 | NINCDS-ADRDA | age, gender, education, MMSE, movement disorders (including EPS), NPS, depression, cerebrovascular disease, heart disease, hypertension, diabetes mellitus | nursing home, death | 73.5^a^ for antipsychotics non-receipt and 72.0^a^ for antipsychotics receipt | 67.4 | 4.3 | 3.7 for antipsychotics non-receipt and 4.1 for antipsychotics receipt | NK |
| Rabins *et al*(35) | 2013 | America | 335 | NINCDS-ADRDA | age, gender | severe AD | 84.3^b^ | NA | 3.53 | NK | 8.4 |
| Nägga *et al*(36) | 2014 | Sweden | 247 | NINCDS-ADRDA | age, MMSE, hypertension | death | 75.2^a^ | 69 | 12.6 | NK | 6.4 |
| Degerman Gunnarsson *et al*(37) | 2014 | Sweden | 196 | DSM-IV, NINCDS-ADRDA | age, APOE ε4, heart disease | rapid cognitive decline, death | 70.0^a^ | 57 | 6 | NK | NK |
| Wattmo *et al*(38) | 2014 | Sweden | 791 | DSM-IV, NINCDS-ADRDA | age, age of onset, gender, education, MMSE, ADL, PSMS, APOE ε4 | death | 76.1^a^  73.0^b^ | 62 | 15 | 3.1 | NK |
| Benedictus *et al*(39) | 2015 | The Netherlands | 333 | NIA-AA, NINCDS-ADRDA | cerebrovascular disease | death | 71.2^a^ | 42 | At least 3 years | NK | NK |
| Wattmo *et al*(40) | 2015 | Sweden | 1021 | DSM-IV, NINCDS-ADRDA | age, age of onset, gender, education, marital status, MMSE, ADL, PSMS, APOE ε4 | death | 76.0^a^, 72.9^b^ for deceased, 71.7^a^, 68.8^b^ for alive | 64.1 | 3.0 | 3.1 for deceased, 3.0 for alive | 6.0 |
| Lin *et al*(41) | 2015 | China | 1438 | ICD 10 code for AD, DSM-IV | gender | CDR progression | subgroup | 77.1 | 1.0 | NK | NK |
| Degerman Gunnarsson *et al*(42) | 2016 | Sweden | 234 | DSM-IV, NINCDS-ADRDA | age, gender, marital status, MMSE | NHP, conversion to moderate dementia, death | 70.0^a^ | 62 | 4.9 | NK | subgroup |
| Nielsen *et al*(43) | 2016 | Denmark | 45894 | ICD 10 code for AD | VRF, somatic comorbidity score, NPS | death | 82.9^a^ | 62.8 | 82.9 | NK | NK |
| Mueller *et al*(44) | 2017 | England | 5473 | ICD 10 code for AD | gender, race, marital status, MMSE, ADL, NPS, depression, cerebrovascular disease | death | subgroup and almost >80.0^a^ | 64 | 3.5 | NK | NK |
| Rhodius-Meester *et al*(45) | 2018 | The Netherlands | 616 | NIA-AA, NINCDS-ADRDA | age, gender, education, smoking, MMSE, ADL, APOE ε4, Aβ_42,_ t-tau, heart disease, hypertension, hyperlipidaemia, diabetes mellitus | death | 66.0^a^ for alive and 69.0^a^ for died | 51 | 4.9 | NK | 4.3 |
| Mueller *et al*(46) | 2018 | England | 2464 | ICD 10 code for AD | age, gender, race, marital status, MMSE, ADL, NPS, depression, heart disease, respiratory disease, cancer, | death | 83.1^a^ for AChEI non-receipt and 80.8^a^ for AChEI receipt | 66.7 | 3.66 | NK | NK |
| Ku *et al*(47) | 2018 | China | 8614 | ICD-9-CM code for AD, NINCDS-ADRDA | gender | death | subgroup and almost >80.0^a^ | 60.8 | subgroup | NK | NK |
| Black *et al*(48) | 2018 | America | 8995 | ICD-9-CM code for AD | gender, race, somatic comorbidity score, NPS, respiratory disease | institutionalization, death | 83.6^a^ | 74.2 | at least 1 year | NK | NK |
| Nielsen *et al*(49) | 2018 | Denmark | 32001 | ICD 10 code for AD | gender, VRF, somatic comorbidity score, NPS | death | subgroup and almost >80.0^b^ | 65 | up to 12 | NK | age at death: 85.26 |
| Giil *et al*(50) | 2018 | Norway | 90 | NINCDS-ADRDA | age, gender, weight loss | death | 75.0^a^ | 73 | 5 | NK | NK |
| Chu *et al*(51) | 2018 | China | 1470 | ICD-9-CM code for AD | age, gender, hypertension, depression, cerebrovascular disease, heart disease, hyperlipidaemia, diabetes mellitus | death | 78.3^a^ | 48.2 | 5.2 | NK | NK |
| Chen *et al*(52) | 2019 | China | 84 | NIA-AA, NINCDS-ADRDA | weight loss | death | 86.6^a^ | 0.71 | 2.1 | NK | 3.5 |
| Linna *et al*(53) | 2019 | Finland | 9204 | ICD 10 code for AD | age, gender, marital status, heart disease, cerebrovascular disease, respiratory disease, diabetes mellitus, cancer | death | 83.0^a^ | 66.8 | 1.8 | NK | NK |
| Boumenir *et al*(54) | 2019 | France | 321 | NIA-AA | age, gender, MMSE, APOE ε4, Aβ_42_, t-tau | institutionalization, death | 71.0^a^ | 62.3 | 3.9 | NK | NK |
| Chen *et al*(55) | 2020 | China | 1754 | ICD-9-CM code for AD | gender, heart disease, cerebrovascular disease, respiratory disease, hypertension, hyperlipidaemia, diabetes mellitus | death | 76.0^a^ for high SES and 79.0^a^ for low SES | 51.1 | 5 | NK | NK |
| de Sousa *et al*(56) | 2020 | Portugal | 79 | DSM-Ⅴ, NIA-AA, NINCDS-ADRDA | weight loss | death | 79.0^a^ | 60 | up to 5 years | NK | NK |
| Zhang *et al*(57) | 2020 | China | 132 | NINCDS-ADRDA | age, age of onset, gender, MMSE, ADL, diabetes mellitus | death | 72.05^a^  69.11^b^ | 56.8 | up to 10 years | 2.95 | 12.1 |
| Wattmo *et al*(58) | 2021 | Sweden | 129 | DSM-IV, NINCDS-ADRDA | age, age of onset, gender, education, marital status, MMSE, ADL, PSMS, APOE ε4, Aβ_42_, t-tau | institutionalization, NHs time, death | subgroup and almost 76.0^a^ | 68.2 | up to 20 years | NK | NK |
| Rajamaki *et al*(59) | 2021 | Finland | 70718 | DSM-IV, NINCDS-ADRDA | gender, NPS, cerebrovascular disease, heart disease, respiratory disease, diabetes mellitus | death | 80.05^a^ | 65.2 | 4.89 | NK | NK |
| Gottesman *et al*(60) | 2021 | America | 212 | NINCDS-ADRDA | age, NPS | functional ability, dependency, cognitive ability | 85.0^a^ | 78.3 | 3..69 | NK | subgroup |
| Liew *et al*(61) | 2021 | multiple countries of NACC | 6221 | NIA-AA, NINCDS-ADRDA | NPS | severe dementia | 78.0^a^ | 54.1 | 3.5 | NK | NK |
| van Loenhoud *et al*(62) | 2022 | The Netherlands | 882 | NIA-AA | education | death | 65.6^a^ | 53.9 | 2.3 | NK | NK |
| Ono *et al*(63) | 2022 | Japan | 39081 | ICD 10 code for AD | gender | death | 83.6^a^ | 67.1 | 2.1 | NK | NK |
| Armstrong *et al*(64) | 2022 | America | 9148 | NINCDS-ADRDA | age, age of onset, gender, race, education, marital status, smoking, MMSE, VRF, somatic comorbidity score | death | 72.0^b^ | 55.9 | 2 | NK | 6 |

Abbreviations: AD, Alzheimer’s disease; ^a^, age at study entry or diagnosis; ^b^, age of onset; NINCDS-ADRDA, the National Institute for Neurological and Communicative Disorders and Stroke and Alzheimer's Disease and Related Disorders Association; NHP, nursing home place; MMSE, The Mini Mental State Examination; ADL, Activity of Daily Living; PSMS, Physical Self-Maintenance Scale; APOE, Apolipoprotein E; t-tau, total tau protein; VRF, vascular risk factors; EPS, extrapyramidal signs; NPS, neuropsychiatric symptoms; BMI, body mass index; SES, socioeconomic status.

Supplementary table 4. Quality assessment and confounding factors of included studies in the meta-analysis

| **Author** | **Year** | **Estimate values** | **NOS** | **Confounding factors** |
| --- | --- | --- | --- | --- |
| Walsh *et al*(1) | 1990 | RR | 6 | age at symptom onset, wandering and falling, behavioural problems and hearing problems |
| Burns *et al*(2) | 1991 | HR | 8 | age, sex, MMSE, type of AD, misidentification, depression |
| Stern *et al*(3) | 1994 | RR | 8 | age, sex, disease severity, and estimated duration of illness at study entry |
| Chui *et al*(4) | 1994 | HR | 6 | sex, education level, age at symptom onset, presence of delusions, hallucinations, agitation, and extrapyramidal signs not caused by neuroleptic medication, and family history of dementia. |
| Stern *et al*(5) | 1995 | RR | 6 | CDR, education, gender, and age at the initial visit |
| Bowen *et al*(6) | 1996 | RR | 8 | age, gender, and severity |
| Heyman *et al*(7) | 1996 | HR | 8 | age at entry, sex, race, marital status, number of years of education, duration of dementia at entry, and scores on ADL, CDR, and MMSE |
| Geerlings *et al*(8) | 1997 | RR | 8 | age, sex, and education |
| Stern *et al*(9) | 1997 | RR | 8 | age, sex, MMSE, EPS, psychotic symptoms, duration of illness |
| Stern *et al*(10) | 1997 | RR | 6 | gender and age and mMMS score at the initial visit |
| Tilvis *et al*(11) | 1998 | RR | 6 | age and gender |
| White *et al*(12) | 1998 | RR | 6 | age, sex, initial weight, stage of AD, education, race, and marital status |
| Claus *et al*(13) | 1998 | HR | 8 | age at entry, sex, education, extrapyramidal signs and vascular risk factors, dementia severity, symptom duration, CAMCOG, MMSE, ADL, and behavioural disturbances |
| Claus *et al*(14) | 1999 | HR | 7 | age at entry, sex and cortical atrophy assessments |
| Larson *et al*(15) | 2004 | HR | 7 | age, sex, ethnicity and medical conditions |
| Scarmeas *et al*(16) | 2005 | RR | 6 | cohort (first or second Predictors cohort), recruitment center, age, sex, education, baseline Columbia MMSE, baseline BDRS, and comorbidity index |
| Suh *et al*(17) | 2005 | RR | 7 | age, severity of dementia, MMSE score and vascular risk factors |
| Waring *et al*(18) | 2005 | HR | 8 | age, sex, race, education, marital status |
| Scarmeas *et al*(19) | 2005 | RR | 7 | cohort, recruitment center; informant status; age at intake in the study; sex; education in years; Columbia MMSE score at initial evaluation; BDRS score at initial evaluation; and the comorbidity index |
| McCann *et al*(20) | 2005 | HR | 8 | participant age, gender, race, time-varying MMSE score, and time-varying adult day care use |
| Carcaillon *et al*(21) | 2007 | RR | 6 | age, sex, at least primary education, MMSE |
| Scarmeas *et al*(22) | 2007 | HR | 7 | cohort, recruitment center, age at intake in the study, sex, education in years, Columbia MMSE score at initial evaluation, BDRS score at initial evaluation, the Comorbidity Index, neuroleptic use, and cholinesterase inhibitor use |
| Bruandet *et al(23)* | 2008 | RR | 8 | age, sex, acetylcholinesterase inhibitor treatment, diabetes, hypertension and vascular lesions visible on MRI |
| Helzner *et al*(24) | 2008 | HR | 7 | ethnicity, sex, education, history of heart disease, hypertension at baseline visit, history of stroke, history of diabetes, study cohort, and follow-up time |
| Mehta *et al*(25) | 2008 | HR | 7 | age, sex, education, ADC, marital status at the time of ADC evaluation, and living situation at the time of ADC evaluation |
| Pavlik *et al*(26) | 2009 | HR | 8 | age at diagnosis, sex, race, years of education, duration of symptoms, baseline severity of disease based on MMSE score, and presence of relevant cerebrovascular disease features on imaging. |
| Henneman *et al*(27) | 2009 | HR | 6 | age, sex, and history of hypertension, diabetes mellitus, hypercholesterolemia, myocardial infarction |
| Hatoum *et al*(28) | 2009 | HR | 6 | age, gender, MMSE, ADL |
| Zhou *et al*(29) | 2010 | HR | 8 | gender, age, and disease stage |
| Musicco *et al*(30) | 2011 | HR | 6 | exposure, sex, comorbidity, and propensity score |
| Wattmo *et al*(31) | 2011 | HR | 7 | gender, age, and living status, ChEI dose, MMSE and IADL score at baseline |
| Rountree *et al*(32) | 2012 | HR | 8 | race, presence or history of medical comorbidities, baseline disease severity, and years of formal education |
| Go *et al*(33) | 2013 | HR | 6 | age of onset, gender, year of diagnosis, history of diabetes, CDR scores, MMSE scores |
| Lopez *et al*(34) | 2013 | HR | 6 | antipsychotic exposure, age, education level, gender, and MMSE scores, present baseline extrapyramidal signs, incident stroke/transient ischemic attack, hypertension, diabetes mellitus, heart disease, aggression, agitation, psychosis, major depression, and dementia medication |
| Rabins *et al*(35) | 2013 | RR | 8 | gender, education, age of dementia onset, NPI score, general medical health |
| Nägga *et al*(36) | 2014 | HR | 7 | age, cognition, frontal atrophy, inflammation |
| Degerman Gunnarsson *et al*(37) | 2014 | HR | 7 | age, education, coronary heart disease/heart failure and mild/moderate dementia at baseline |
| Wattmo *et al*(38) | 2014 | HR | 6 | gender, age at start of ChEI treatment, years of education, number of APOE ε4 alleles, age at onset of AD, mMMS, IADL and PSMS scores, number of concomitant medications and antihypertensives/cardiac therapy at baseline |
| Benedictus *et al*(39) | 2015 | HR | 7 | age, sex, MMSE score, vascular risk factors, and the presence of white matter hyperintensities and lacunes |
| Wattmo *et al*(40) | 2015 | HR | 6 | age, sex, lipid-lowering agents, anxiolytics/sedative/hypnotics, ADAS-cog score, number of concomitant medications |
| Lin *et al*(41) | 2015 | HR | 7 | age, sex, hypertension, and diabetes |
| Degerman Gunnarsson *et al*(42) | 2016 | HR | 7 | age, sex, living condition, education level, baseline mild to moderate dementia stage, and MMSE score |
| Nielsen *et al*(43) | 2016 | HR | 7 | age, gender, severity of AD, psychiatric comorbidity, somatic comorbidity and cardiovascular risk factors. |
| Mueller *et al*(44) | 2017 | HR | 6 | age, sex, ethnicity, marital status, deprivation score at dementia diagnosis and MMSE |
| Rhodius-Meester *et al*(45) | 2018 | HR | 8 | age, sex, MMSE and duration of complaints |
| Mueller *et al*(46) | 2018 | HR | 7 | age and gender |
| Ku *et al*(47) | 2018 | HR | 6 | age, sex, CCI, the level of urbanization and annual number of ambulatory care visits |
| Black *et al*(48) | 2018 | HR | 7 | age, race, gender, geographic region, Charlson Comorbidity Index score, baseline all-cause health care utilization, baseline diagnostic tests (CT and MRI), and prevalence of individual comorbidities |
| Nielsen *et al*(49) | 2018 | HR | 6 | severity of NPS, psychiatric comorbidity, somatic comorbidity, and cardiovascular risk factors |
| Giil *et al*(50) | 2018 | HR | 8 | poor renal function, a low body mass index, Trail Making Test A scores, depression and comorbidity |
| Chu *et al*(51) | 2018 | HR | 6 | age, sex, AchEIs, hyperlipidaemia, CCI, major depression, and delirium |
| Chen *et al*(52) | 2019 | HR | 6 | age, gender, and years of education |
| Linna *et al*(53) | 2019 | HR | 6 | age, sex, comorbidities, housing status, income, income decile, single-living status and caregiver help |
| Boumenir *et al*(54) | 2019 | HR | 8 | age, sex, MMSE, level of education, APOE ε4 status, collection tube for CSF, CSF Aβ_42_, and CSF tau |
| Chen *et al*(55) | 2020 | HR | 6 | sex, age, urbanization, area of residence, medical center, district, and regional |
| de Sousa *et al*(56) | 2020 | HR | 8 | sex, age, marital status, education, and MMSE |
| Zhang *et al*(57) | 2020 | HR | 7 | age and sex |
| Wattmo *et al*(58) | 2021 | HR | 7 | sex, age at baseline, the clinician’s estimation of age at onset, years of education, and APOE genotype |
| Rajamaki *et al*(59) | 2021 | HR | 6 | age, sex and socioeconomic position |
| Gottesman *et al*(60) | 2021 | HR | 8 | sex, age at study entry, ethnicity, level of education in years, baseline scale performance in BDRS, MMSE, or Dependence |
| Liew *et al*(61) | 2021 | HR | 7 | age, sex, ethnicity, years of education, APOE ε4 genotype, MMSE score, CDR sum of boxes score, use of cognitive enhancers, use of antidepressants, use of antipsychotics and use of sedatives |
| van Loenhoud *et al*(62) | 2022 | HR | 7 | disease stage, age, sex, whole brain gray matter atrophy, and MRI field strength |
| Ono *et al*(63) | 2022 | HR | 8 | age, sex, Charlson comorbidity index, and antidementia drug use |
| Armstrong *et al*(64) | 2022 | HR | 6 | age at symptom onset, sex, race, ethnicity, dementia type, having multiple dementia diagnoses, type of residence, comorbidity score, cardiovascular risk index, years of smoking, number of medications, MMSE, CDR-SB, NPI-Q, and GDS |

Abbreviations: AD, Alzheimer’s disease; RR, relative risk; HR, hazard ratio; NOS, Newcastle-Ottawa Scale; MMSE/mMMS, The Mini Mental State Examination; CDR, Clinical Dementia Rating; CAMCOG, The Cambridge Cognition Examination; ADL, Activity of Daily Living; BDRS, Blessed Dementia Rating Scale; MRI, Magnetic Resonance Imaging; ADC, AD centers; ChEI, cholinesterase inhibitor; IADL, Instrumental Activities of Daily Living; NPI, Neuropsychiatric Inventory; PSMS, Physical Self-Maintenance Scale; APOE, Apolipoprotein E; ADAS-cog, Alzheimer's Disease Assessment Scale–cognitive subscale; CCI, Charlson Comorbidity Index; CT, Computed Tomography; CSF, cerebrospinal fluid; Aβ, β-amyloid.

Supplementary table 5. Meta-analysis of potential factors for survival in patients with AD

| **Prognostic factors** | **Number of studies** | **Pooled HR and 95% CI** | **P value** | **I^2^** |
| --- | --- | --- | --- | --- |
| **Individual features (7)** |  |  |  |  |
| age (per year increase) | 27 | 1.05 (1.04-1.07) | <0.001 | 92.2% |
| age of onset (per year increase) | 6 | 1.03 (1.01-1.05) | 0.003 | 73.9% |
| gender (ref: female) | 37 | 1.58 (1.49-1.68) | <0.001 | 84.8% |
| race (ref: none-white) | 8 | 1.36 (1.21-1.53) | <0.001 | 75.8% |
| education (per year increase) | 14 | 1.00 (0.98-1.02) | 0.848 | 67.0% |
| marital status (ref: cohabiting) | 10 | 1.07 (0.97-1.19) | 0.167 | 59.2% |
| smoking | 4 | 1.00 (1.00-1.01) | <0.001 | 0.0% |
| **Clinical manifestations or comorbidities (13)** |  |  |  |  |
| hyperlipidaemia | 4 | 0.69 (0.59-0.80) | <0.001 | 0.0% |
| cancer | 3 | 2.07 (1.17-3.67) | 0.013 | 92.9% |
| movement disorders (including EPS) | 7 | 1.60 (1.32-1.93) | <0.001 | 58.4% |
| NPS (including behavioural problems) | 14 | 1.16 (1.08-1.24) | <0.001 | 94.2% |
| depression | 7 | 1.12 (1.03-1.22) | 0.011 | 26.4% |
| heart disease | 12 | 1.24 (1.11-1.37) | <0.001 | 76.7% |
| cerebrovascular disease | 12 | 1.30 (1.20-1.41) | <0.001 | 61.6% |
| respiratory disease | 5 | 1.23 (1.19-1.27) | <0.001 | 11.5% |
| somatic comorbidity score | 4 | 1.24 (1.06-1.44) | 0.007 | 99.6% |
| diabetes mellitus | 12 | 1.30 (1.15-1.48) | <0.001 | 75.1% |
| hypertension | 9 | 1.19 (1.00-1.41) | 0.051 | 76.8% |
| wandering or falling | 4 | 1.39 (0.95-2.06) | 0.093 | 49.7% |
| VRF | 5 | 1.02 (0.93-1.13) | 0.632 | 87.7% |
| **Rating scales (3)** |  |  |  |  |
| MMSE scores (per point increase) | 15 | 0.93 (0.91-0.95) | <0.001 | 85.6% |
| ADL scores (per point increase) | 10 | 1.11 (1.07-1.16) | <0.001 | 93.4% |
| PSMS scores (per point increase) | 4 | 1.09 (1.07-1.10) | <0.001 | 36.1% |
| **Biomarkers (3)** |  |  |  |  |
| APOE ε4 carrier | 10 | 0.94 (0.78-1.14) | 0.532 | 66.7% |
| Aβ_42_ | 3 | 1.09 (0.91-1.32) | 0.356 | 78.0% |
| t-tau | 3 | 1.00 (1.00-1.01) | 0.046 | 0.0% |
| **Secondary outcome (9)** |  |  |  |  |
| movement disorders (including EPS) | 3 | 1.76 (1.11-2.79) | 0.001 | 74.7% |
| NPS | 7 | 1.35 (1.25-1.46) | <0.001 | 2.4% |
| MMSE scores (per point increase) | 8 | 0.93 (0.90-0.96) | <0.001 | 82.7% |
| age (per year increase) | 7 | 1.02 (0.98-1.06) | 0.389 | 94.8% |
| age of onset (per year increase) | 4 | 0.98 (0.92-1.05) | 0.592 | 76.4% |
| gender (ref: female) | 10 | 0.92 (0.81-1.04) | 0.165 | 91.7% |
| marital status (ref: cohabiting) | 3 | 1.67 (0.66-4.26) | 0.281 | 85.7% |
| ADL scores (per point increase) | 3 | 1.05 (0.96-1.16) | 0.276 | 95.4% |
| APOE ε4 carrier | 3 | 0.93 (0.72-1.19) | 0.562 | 0.0% |

Abbreviations: AD, Alzheimer’s disease; ref, reference; HR, hazard ratio; CI, confidence intervals; MMSE, The Mini Mental State Examination; ADL, Activity of Daily Living; PSMS, Physical Self-Maintenance Scale; EPS, extrapyramidal signs; NPS, neuropsychiatric symptoms; VRF, vascular risk factors; APOE, Apolipoprotein E; Aβ, β-amyloid; t-tau, total tau protein.

Supplementary figure 1. Forest plot of demographic features as prognostic factors in AD patients.


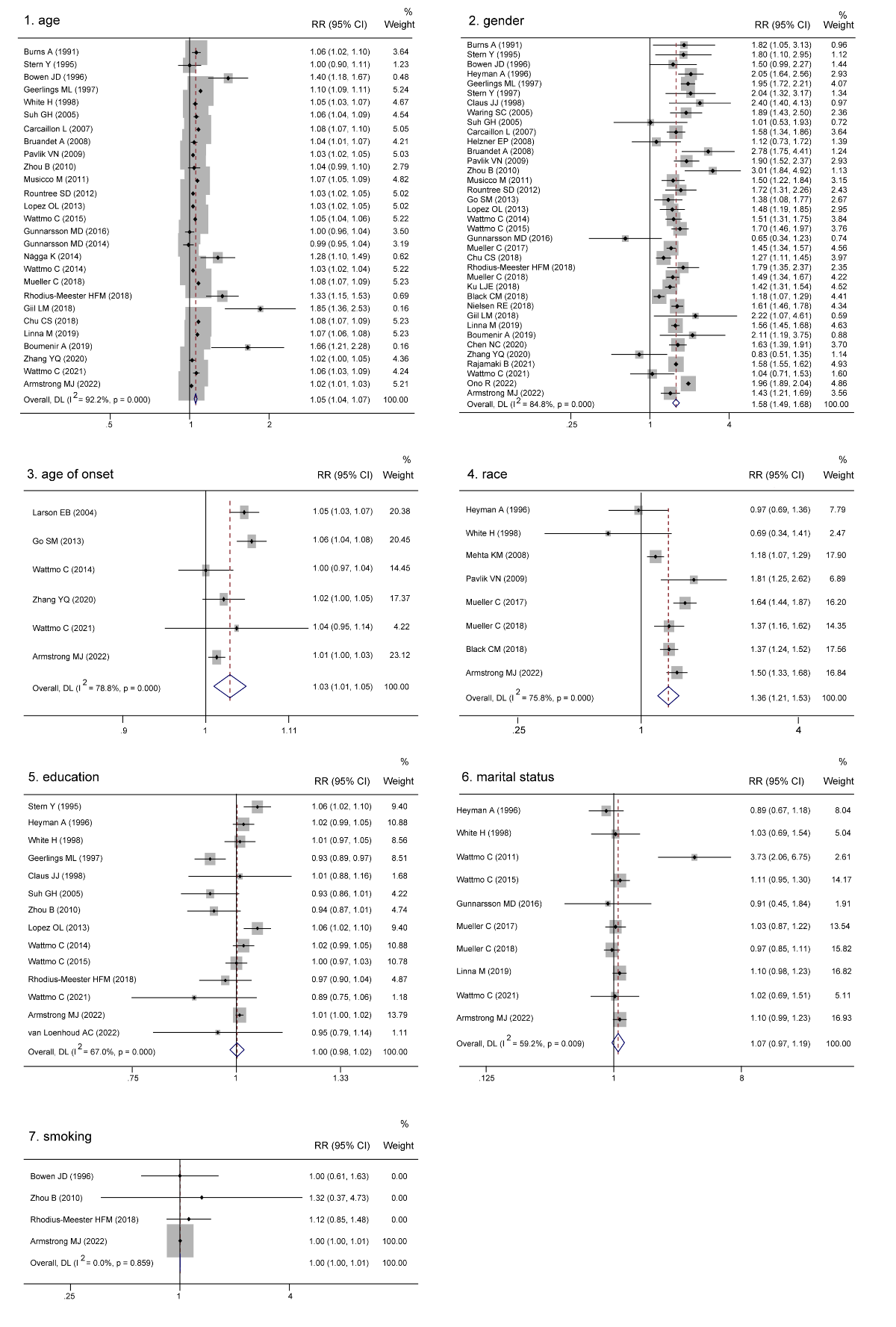


Abbreviations: AD, Alzheimer’s disease; RR, relative risk; CI, confidence intervals.

Supplementary figure 2-A. Forest plot of clinical features or comorbidities as prognostic factors in AD patients.


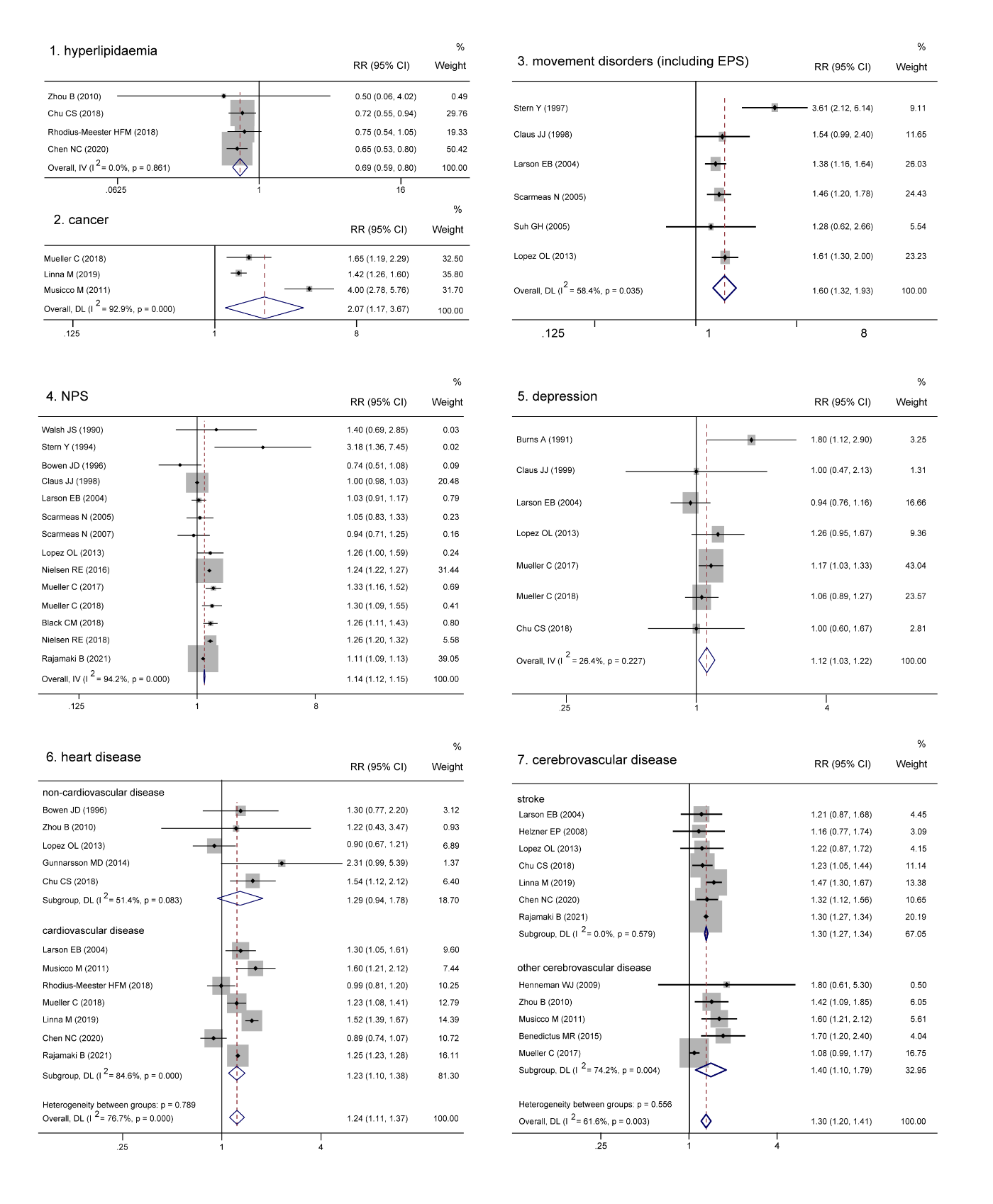


Abbreviations: AD, Alzheimer’s disease; RR, relative risk; CI, confidence intervals; EPS, extrapyramidal signs; NPS, neuropsychiatric symptoms.

Supplementary figure 2-B. Forest plot of clinical features or comorbidities as prognostic factors in AD patients.

**
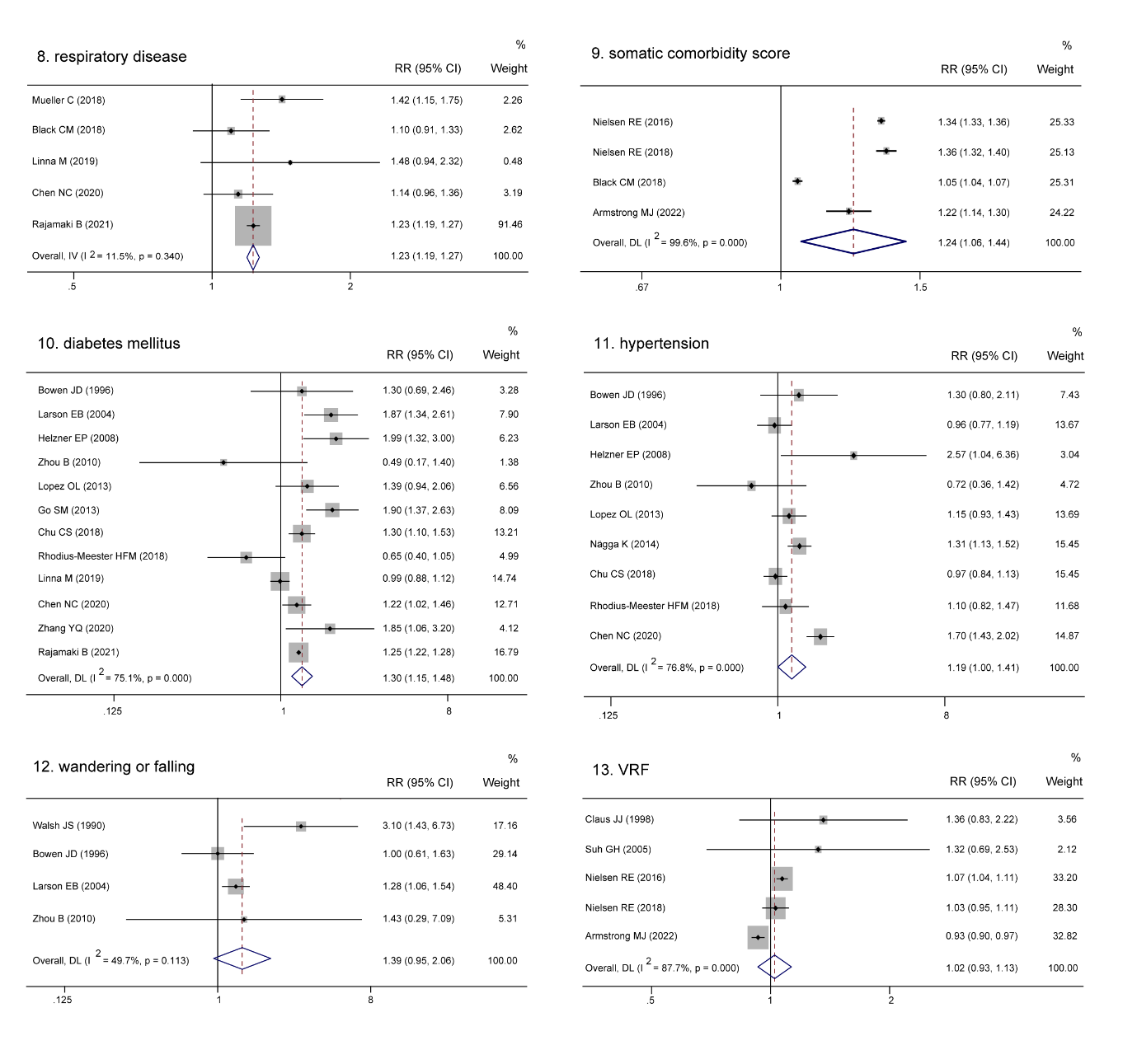
**

Abbreviations: AD, Alzheimer’s disease; RR, relative risk; CI, confidence intervals; VRF, vascular risk factors.

Supplementary figure 3. Forest plot of rating scales as prognostic factors in AD patients.


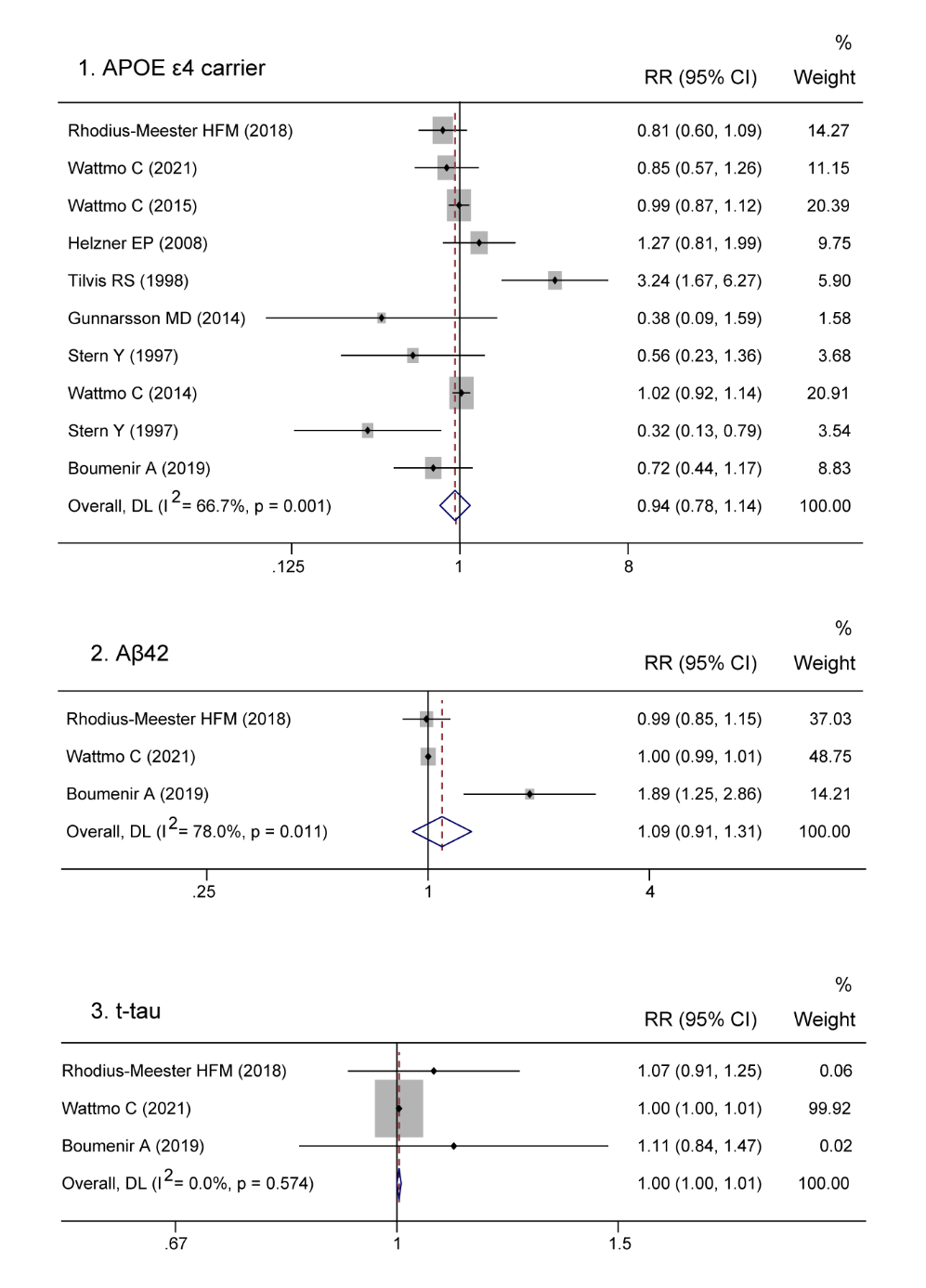


Abbreviations: AD, Alzheimer’s disease; RR, relative risk; CI, confidence intervals; APOE, Apolipoprotein E; Aβ, β-amyloid; t-tau, total tau protein.

Supplementary figure 4. Forest plot of biomarkers as prognostic factors in AD patients.


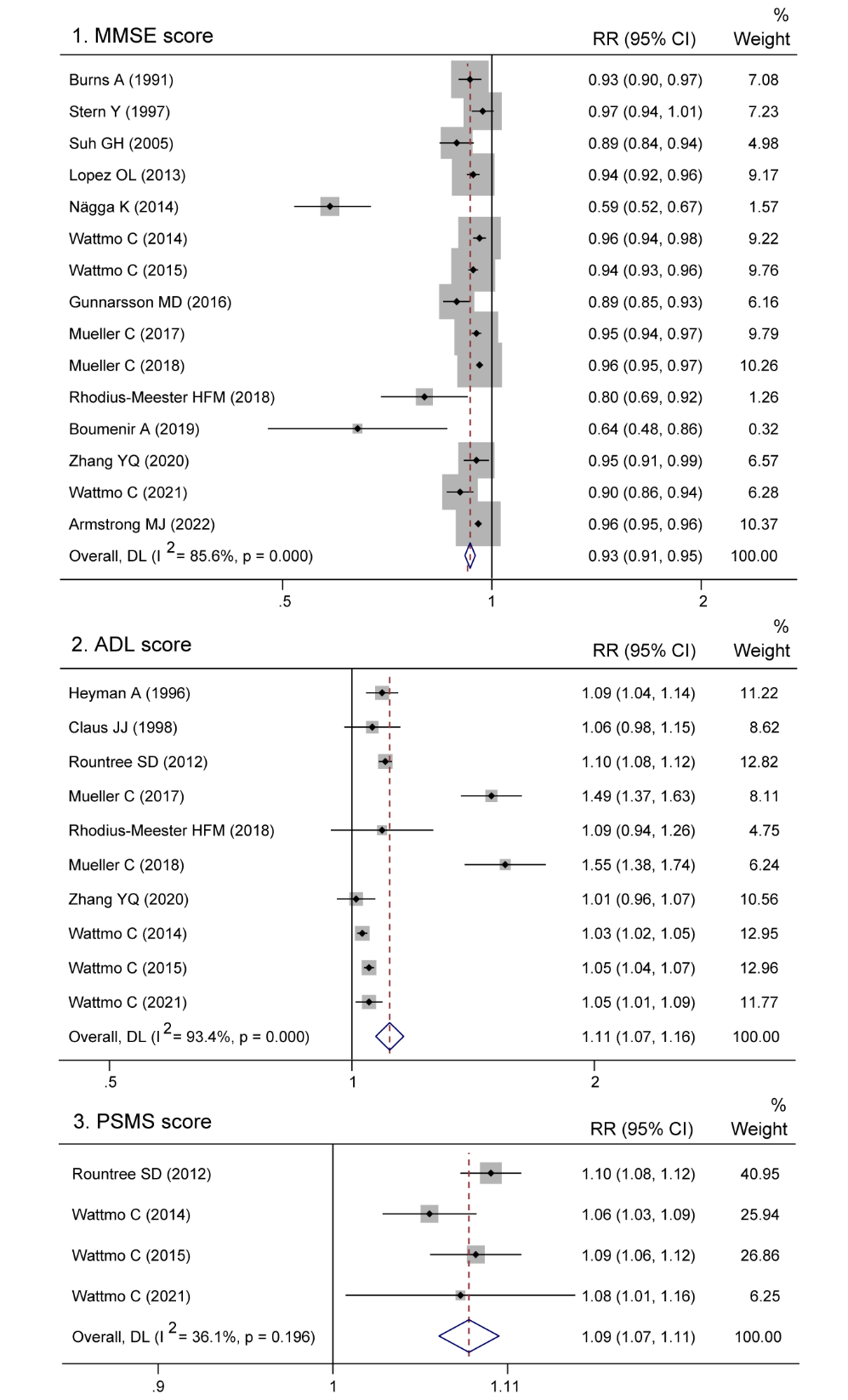


Abbreviations: AD, Alzheimer’s disease; RR, relative risk; CI, confidence intervals; MMSE, [The Mini Mental State Examination;](https://www.brandeis.edu/roybal/docs/MMSE_website.pdf) ADL, Activity of Daily Living; PSMS, Physical Self-Maintenance Scale.

Supplementary figure 5. Forest plot for secondary outcome of prognostic factors in AD patients.

**
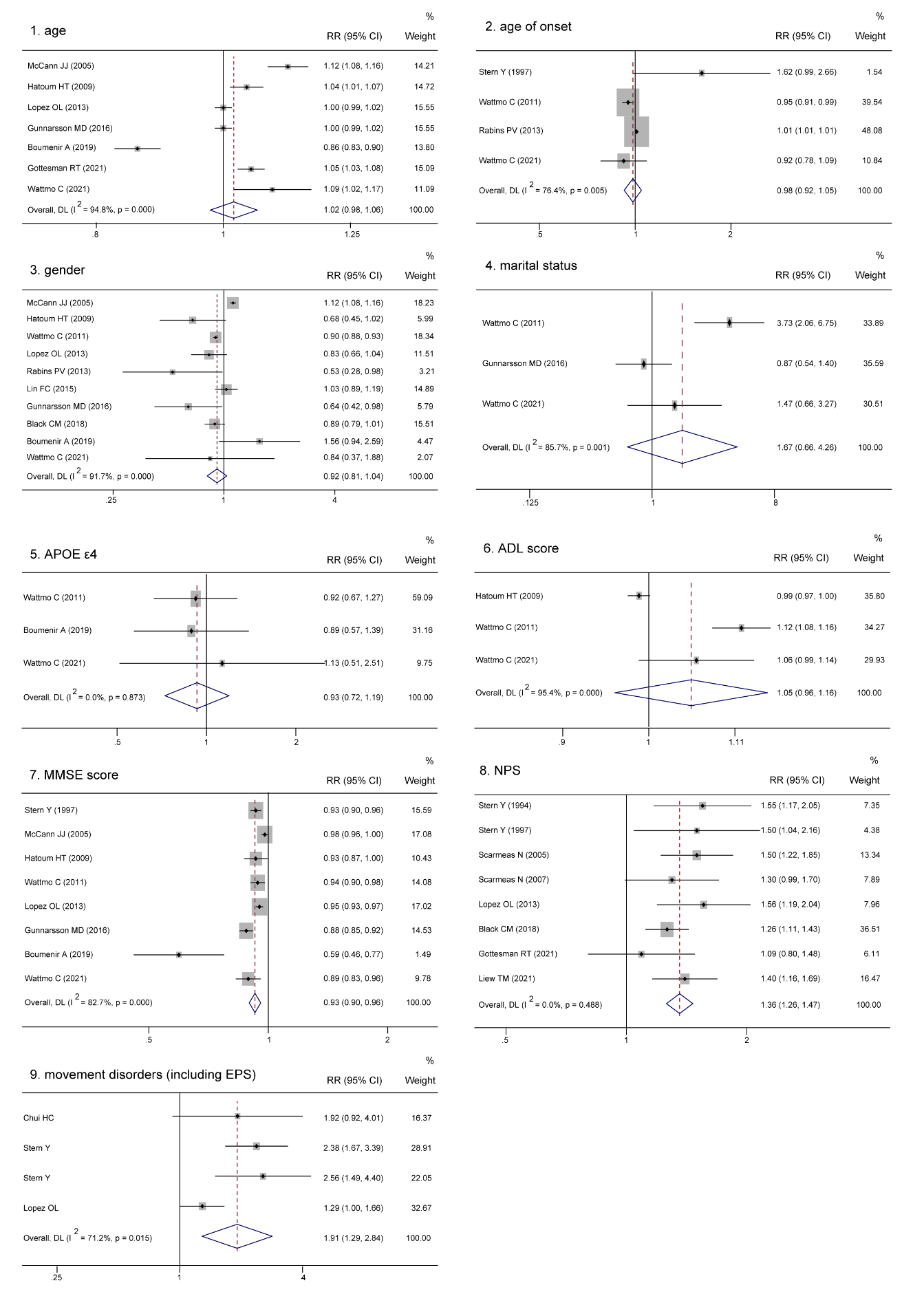
**

Abbreviations: AD, Alzheimer’s disease; RR, relative risk; CI, confidence intervals; APOE, Apolipoprotein E; ADL, Activity of Daily Living; MMSE, [The Mini Mental State Examination;](https://www.brandeis.edu/roybal/docs/MMSE_website.pdf) NPS, neuropsychiatric symptoms; EPS, extrapyramidal signs.

Supplementary figure 6-A. Forest plot for subgroup analysis of prognostic factors in AD patients.


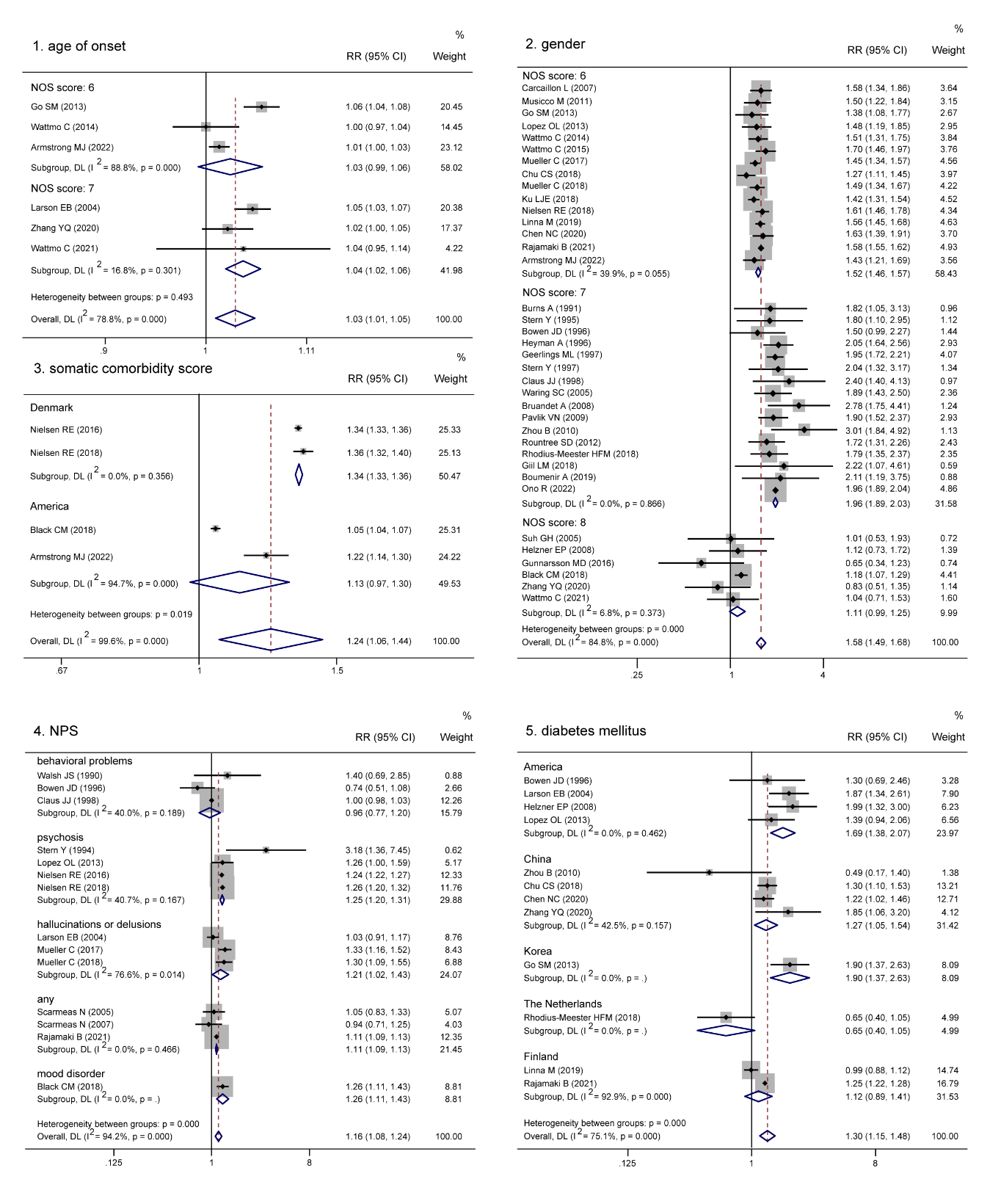


Abbreviations: AD, Alzheimer’s disease; RR, relative risk; CI, confidence intervals; NPS, neuropsychiatric symptoms.

Supplementary figure 6-B. Forest plot for subgroup analysis of prognostic factors in AD patients.


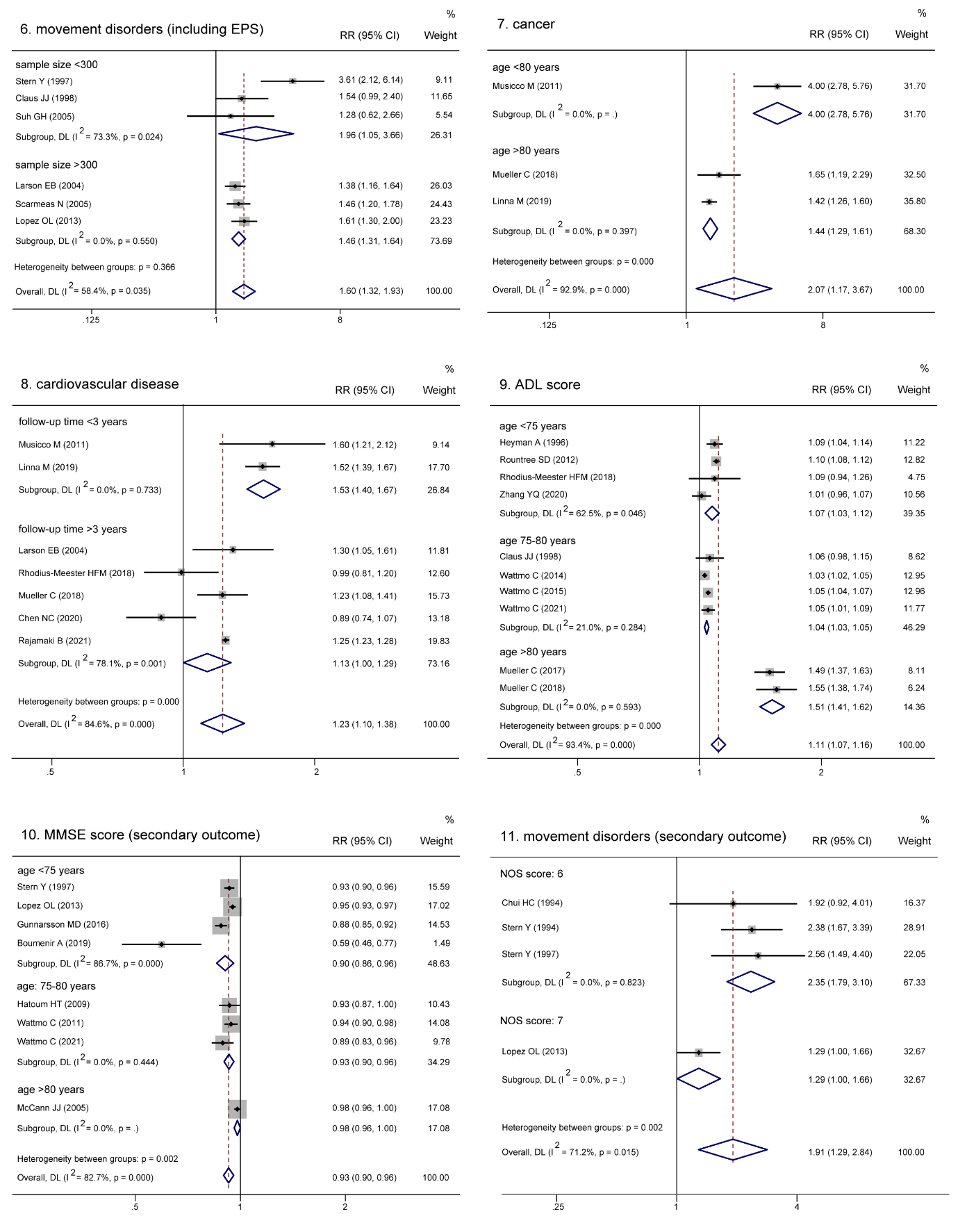


Abbreviations: AD, Alzheimer’s disease; RR, relative risk; CI, confidence intervals; EPS, extrapyramidal signs; ADL, Activity of Daily Living; MMSE, [The Mini Mental State Examination](https://www.brandeis.edu/roybal/docs/MMSE_website.pdf).

Supplementary figure 7-A. Sensitive analysis of prognostic factors in AD patients.

**
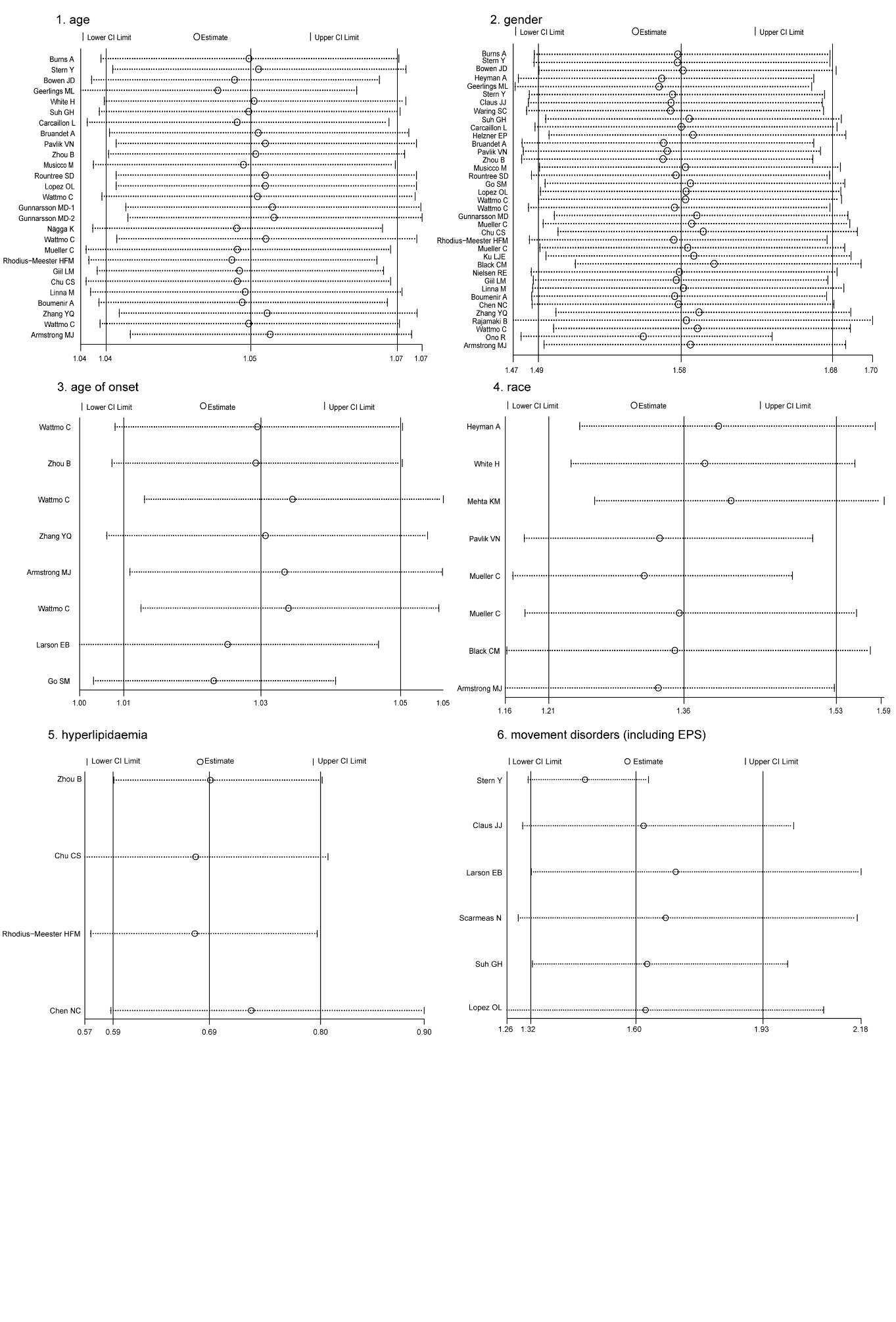
**

Abbreviations: AD, Alzheimer’s disease; EPS, extrapyramidal signs.

Supplementary figure 7-B. Sensitive analysis of prognostic factors in AD patients.


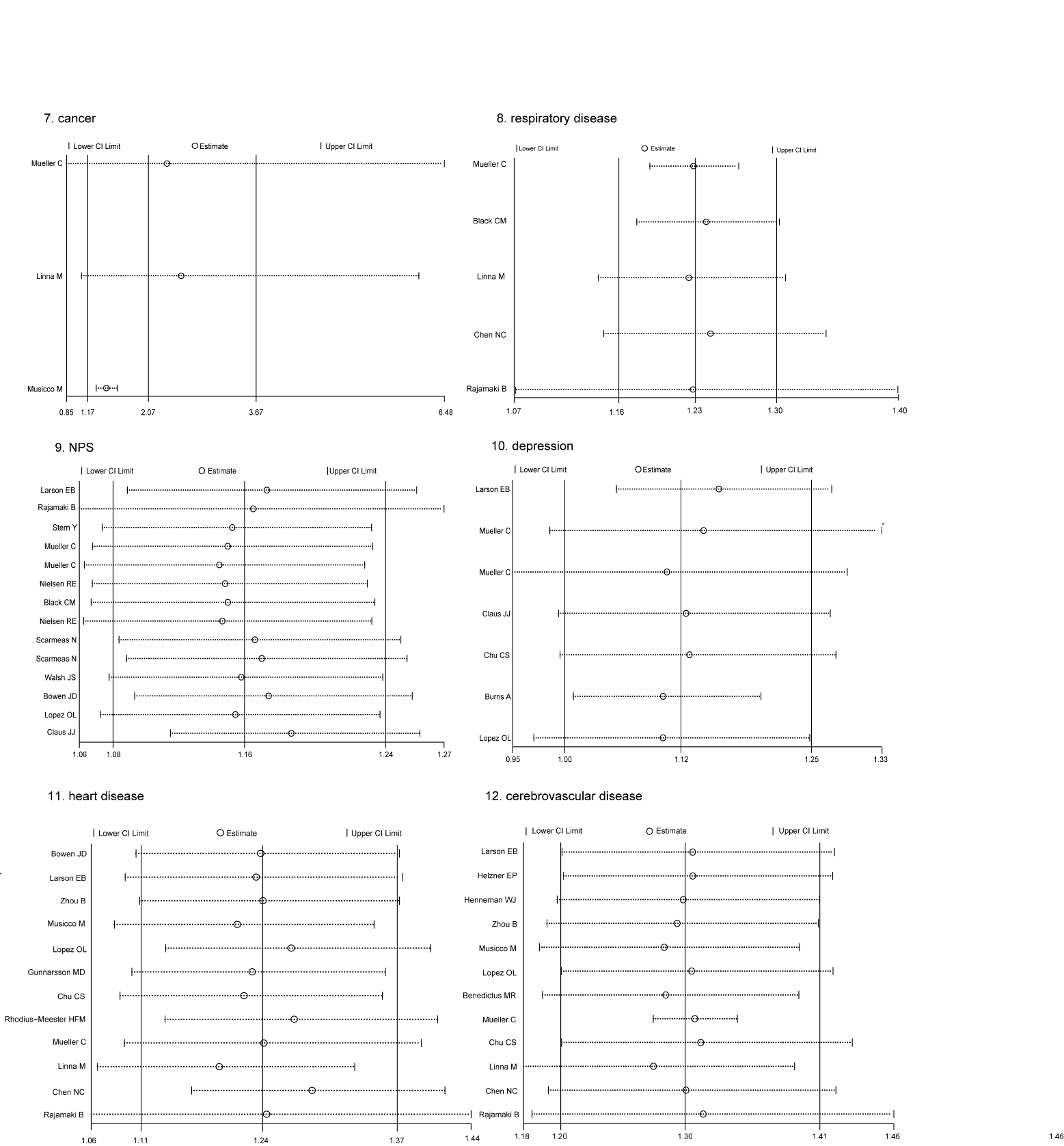


Abbreviations: AD, Alzheimer’s disease; NPS, neuropsychiatric symptoms.

Supplementary figure 7-C. Sensitive analysis of prognostic factors in AD patients.


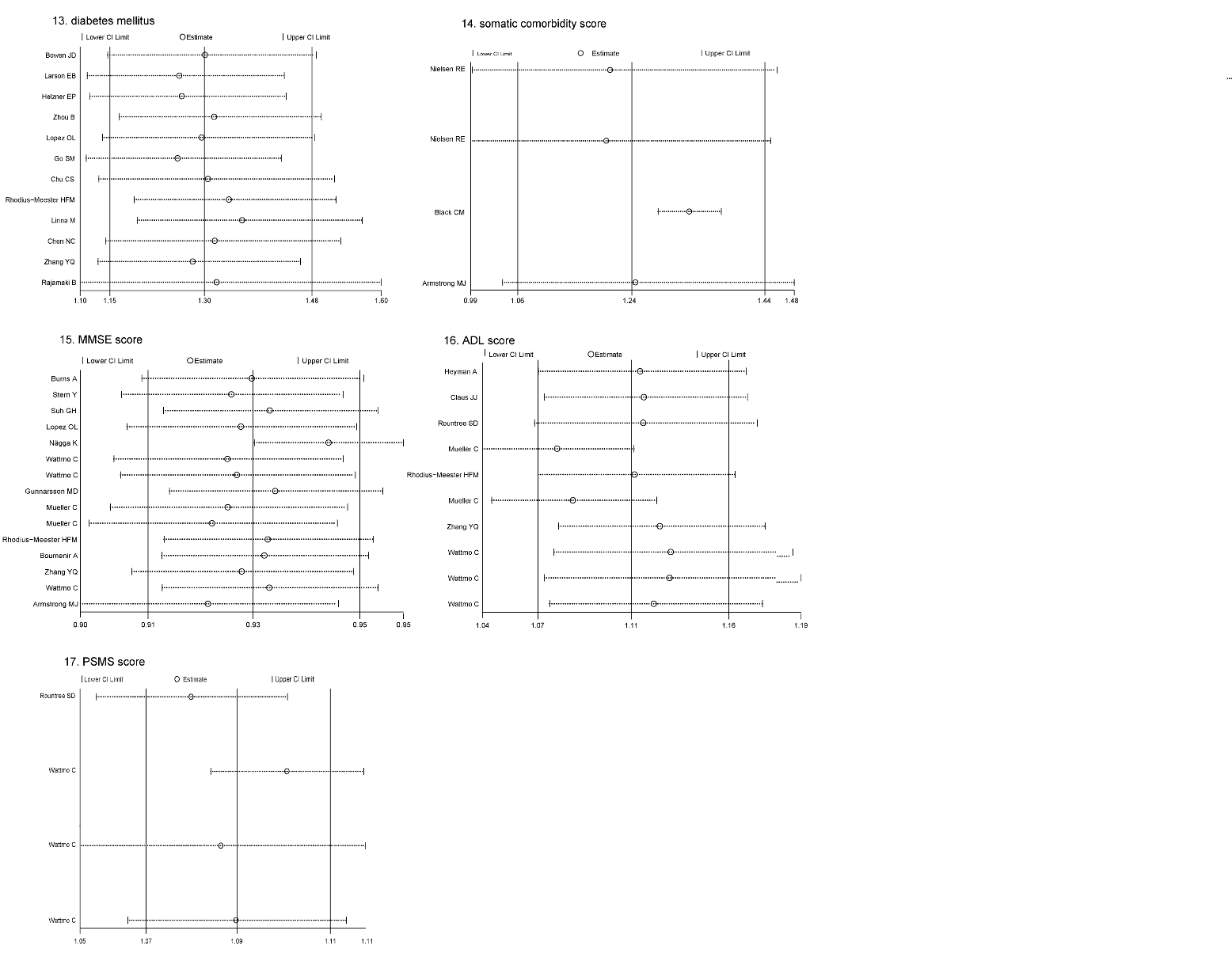


Abbreviations: AD, Alzheimer’s disease; MMSE, The Mini Mental State Examination; ADL, Activity of Daily Living; PSMS, Physical Self-Maintenance Scale.

Supplementary figure 8. Sensitive analysis for secondary outcome of prognostic factors in AD patients.


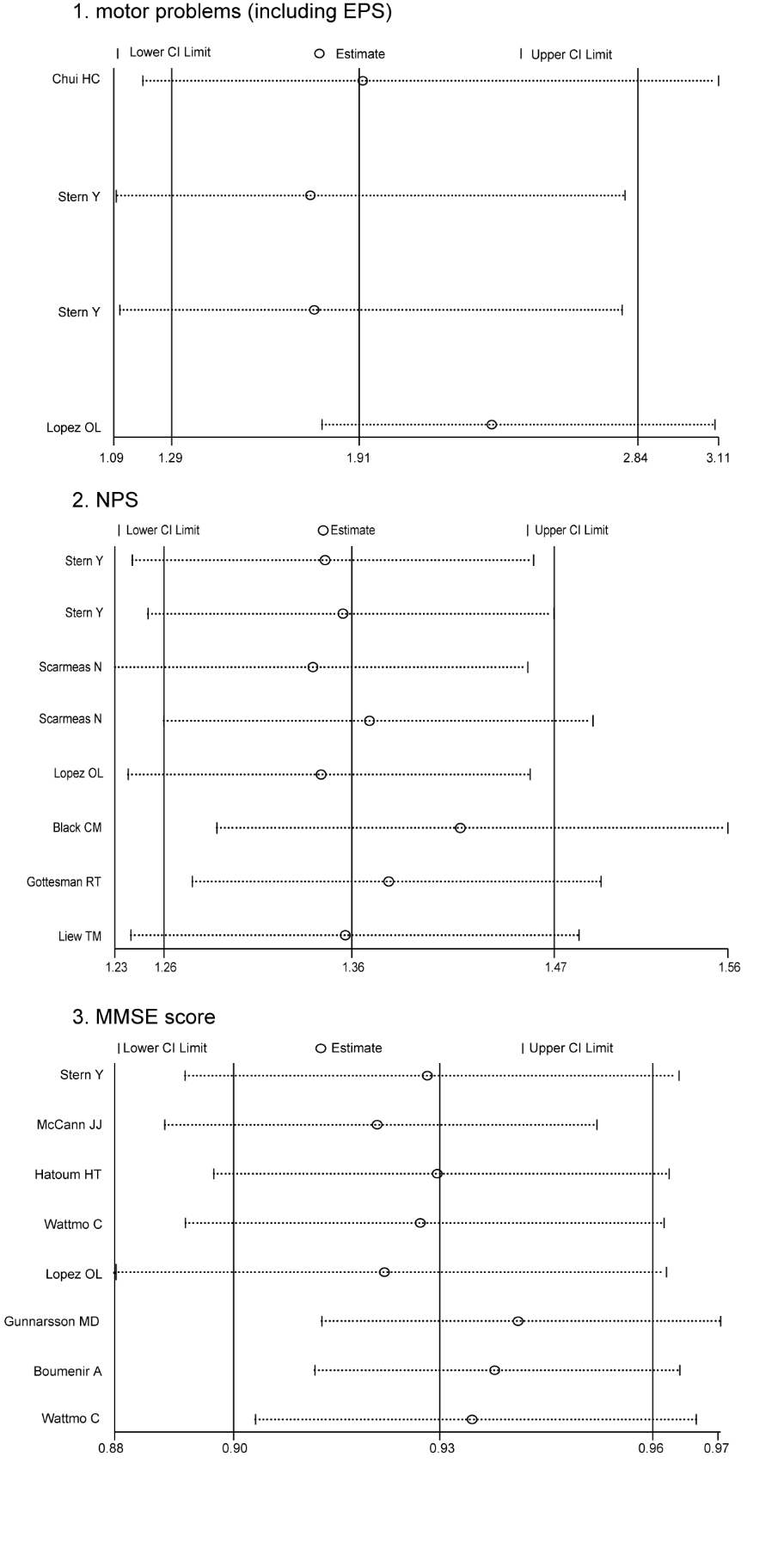


Abbreviations: AD, Alzheimer’s disease; EPS, extrapyramidal signs; NPS, neuropsychiatric symptoms; MMSE, The Mini Mental State Examination.

Reference

1. Walsh JS, Welch HG, Larson EB. Survival of outpatients with Alzheimer-type dementia. Ann Intern Med. 1990;113(6):429-34.

2. Burns A, Lewis G, Jacoby R, Levy R. Factors affecting survival in Alzheimer's disease. Psychological Medicine. 1991;21(2):363-70.

3. Stern Y, Albert M, Brandt J, Jacobs DM, Tang MX, Marder K, et al. Utility of extrapyramidal signs and psychosis as predictors of cognitive and functional decline, nursing home admission, and death in Alzheimer's disease: Prospective analyses from the predictors study. Neurology. 1994;44(12):2300-7.

4. Chui HC, Lyness SA, Sobel E, Schneider LS. Extrapyramidal signs and psychiatric symptoms predict faster cognitive decline in Alzheimer's disease. Archives of Neurology. 1994;51(7):676-81.

5. Stern Y, Tang MX, Denaro J, Mayeux R. Increased risk of mortality in Alzheimer's disease patients with more advanced educational and occupational attainment. Ann Neurol. 1995;37(5):590-5.

6. Bowen JD, Malter AD, Sheppard L, Kukull WA, McCormick WC, Teri L, et al. Predictors of mortality in patients diagnosed with probable Alzheimer's disease. Neurology. 1996;47(2):433-9.

7. Heyman A, Peterson B, Fillenbaum G, Pieper C. The consortium to establish a registry for Alzheimer's disease (CERAD). Part XIV: Demographic and clinical predictors of survival in patients with Alzheimer's disease. Neurology. 1996;46(3):656-60.

8. Geerlings MI, Deeg DJH, Schmand B, Lindeboom J, Jonker C. Increased risk of mortality in Alzheimer's disease patients with higher education? A replication study. Neurology. 1997;49(3):798-802.

9. Stern Y, Tang MX, Albert MS, Brandt J, Jacobs DM, Bell K, et al. Predicting time to nursing home care and death in individuals with Alzheimer disease. Journal of the American Medical Association. 1997;277(10):806-12.

10. Stern Y, Brandt J, Albert M, Jacobs DM, Liu X, Bell K, et al. The absence of an apolipoprotein epsilon4 allele is associated with a more aggressive form of Alzheimer's disease. Ann Neurol. 1997;41(5):615-20.

11. Tilvis RS, Strandberg TE, Juva K. Apolipoprotein E phenotypes, dementia and mortality in a prospective population sample. J Am Geriatr Soc. 1998;46(6):712-5.

12. White H, Pieper C, Schmader K. The association of weight change in Alzheimer's disease with severity of disease and mortality: a longitudinal analysis. J Am Geriatr Soc. 1998;46(10):1223-7.

13. Claus JJ, van Gool WA, Teunisse S, Walstra GJ, Kwa VI, Hijdra A, et al. Predicting survival in patients with early Alzheimer's disease. Dement Geriatr Cogn Disord. 1998;9(5):284-93.

14. Claus JJ, Ongerboer De Visser BW, Walstra GJM, Hijdra A, Verbeeten Jr B, Van Gool WA. Quantitative spectral electroencephalography in predicting survival in patients with early Alzheimer disease. Archives of Neurology. 1998;55(8):1105-11.

15. Larson EB, Shadlen MF, Wang L, McCormick WC, Bowen JD, Teri L, et al. Survival after Initial Diagnosis of Alzheimer Disease. Annals of Internal Medicine. 2004;140(7):501-9+I26.

16. Scarmeas N, Albert M, Brandt J, Blacker D, Hadjigeorgiou G, Papadimitriou A, et al. Motor signs predict poor outcomes in Alzheimer disease. Neurology. 2005;64(10):1696-703.

17. Suh GH, Yeon BK, Shah A, Lee JY. Mortality in Alzheimer's disease: A comparative prospective Korean study in the community and nursing homes. International Journal of Geriatric Psychiatry. 2005;20(1):26-34.

18. Waring SC, Doody RS, Pavlik VN, Massman PJ, Chan W. Survival among patients with dementia from a large multi-ethnic population. Alzheimer Dis Assoc Disord. 2005;19(4):178-83.

19. Scarmeas N, Brandt J, Albert M, Hadjigeorgiou G, Papadimitriou A, Dubois B, et al. Delusions and hallucinations are associated with worse outcome in Alzheimer disease. Arch Neurol. 2005;62(10):1601-8.

20. McCann JJ, Hebert LE, Li Y, Wolinsky FD, Gilley DW, Aggarwal NT, et al. The effect of adult day care services on time to nursing home placement in older adults with Alzheimer's disease. Gerontologist. 2005;45(6):754-63.

21. Carcaillon L, Pérès K, Péré JJ, Helmer C, Orgogozo JM, Dartigues JF. Fast cognitive decline at the time of dementia diagnosis: A major prognostic factor for survival in the community. Dementia and Geriatric Cognitive Disorders. 2007;23(6):439-45.

22. Scarmeas N, Brandt J, Blacker D, Albert M, Hadjigeorgiou G, Dubois B, et al. Disruptive behavior as a predictor in Alzheimer disease. Archives of Neurology. 2007;64(12):1755-61.

23. Bruandet A, Richard F, Bombois S, Maurage CA, Masse I, Amouyel P, et al. Cognitive decline and survival in Alzheimer's disease according to education level. Dementia and Geriatric Cognitive Disorders. 2008;25(1):74-80.

24. Helzner EP, Scarmeas N, Cosentino S, Tang MX, Schupf N, Stern Y. Survival in Alzheimer disease: a multiethnic, population-based study of incident cases. Neurology. 2008;71(19):1489-95.

25. Mehta KM, Yaffe K, Pérez-Stable EJ, Stewart A, Barnes D, Kurland BF, et al. Race/ethnic differences in AD survival in US Alzheimer's Disease Centers. Neurology. 2008;70(14):1163-70.

26. Pavlik VN, Doody RS, Rountree SD, Darby EJ. Vitamin E Use Is Associated with Improved Survival in an Alzheimer's Disease Cohort. Dementia and Geriatric Cognitive Disorders. 2009;28(6):536-40.

27. Henneman WJ, Sluimer JD, Cordonnier C, Baak MM, Scheltens P, Barkhof F, et al. MRI biomarkers of vascular damage and atrophy predicting mortality in a memory clinic population. Stroke. 2009;40(2):492-8.

28. Hatoum HT, Thomas SK, Lin SJ, Lane R, Bullock R. Predicting time to nursing home placement based on activities of daily living scores--a modelling analysis using data on Alzheimer's disease patients receiving rivastigmine or donepezil. J Med Econ. 2009;12(2):98-103.

29. Zhou B, Zhao Q, Teramukai S, Ding D, Guo Q, Fukushima M, et al. Executive function predicts survival in Alzheimer disease: A study in Shanghai. Journal of Alzheimer's Disease. 2010;22(2):673-82.

30. Musicco M, Palmer K, Russo A, Caltagirone C, Adorni F, Pettenati C, et al. Association between prescription of conventional or atypical antipsychotic drugs and mortality in older persons with Alzheimer's disease. Dementia and Geriatric Cognitive Disorders. 2011;31(3):218-24.

31. Wattmo C, Wallin AK, Londos E, Minthon L. Risk factors for nursing home placement in Alzheimer's disease: a longitudinal study of cognition, ADL, service utilization, and cholinesterase inhibitor treatment. Gerontologist. 2011;51(1):17-27.

32. Rountree SD, Chan W, Pavlik VN, Darby EJ, Doody RS. Factors that influence survival in a probable Alzheimer disease cohort. Alzheimer's Research and Therapy. 2012;4(3).

33. Go SM, Lee KS, Seo SW, Chin J, Kang SJ, Moon SY, et al. Survival of alzheimer's disease patients in Korea. Dementia and Geriatric Cognitive Disorders. 2013;35(3-4):219-28.

34. Lopez OL, Becker JT, Chang YF, Sweet RA, Aizenstein H, Snitz B, et al. The long-term effects of conventional and atypical antipsychotics in patients with probable Alzheimer's disease. Am J Psychiatry. 2013;170(9):1051-8.

35. Rabins PV, Schwartz S, Black BS, Corcoran C, Fauth E, Mielke M, et al. Predictors of progression to severe Alzheimer's disease in an incidence sample. Alzheimers Dement. 2013;9(2):204-7.

36. Nägga K, Wattmo C, Zhang Y, Wahlund LO, Palmqvist S. Cerebral inflammation is an underlying mechanism of early death in Alzheimer's disease: A 13-year cause-specific multivariate mortality study. Alzheimer's Research and Therapy. 2014;6(4).

37. Degerman Gunnarsson M, Lannfelt L, Ingelsson M, Basun H, Kilander L. High tau levels in cerebrospinal fluid predict rapid decline and increased dementia mortality in Alzheimer's disease. Dementia and Geriatric Cognitive Disorders. 2014;37(3-4):196-206.

38. Wattmo C, Londos E, Minthon L. Risk factors that affect life expectancy in Alzheimer's disease: a 15-year follow-up. Dement Geriatr Cogn Disord. 2014;38(5-6):286-99.

39. Benedictus MR, Prins ND, Goos JDC, Scheltens P, Barkhof F, Van Der Flier WM. Microbleeds, Mortality, and Stroke in Alzheimer Disease The MISTRAL Study. JAMA Neurology. 2015;72(5):539-45.

40. Wattmo C, Londos E, Minthon L. Longitudinal associations between survival in Alzheimer's disease and cholinesterase inhibitor use, progression, and community-based services. Dementia and Geriatric Cognitive Disorders. 2015;40(5-6):297-310.

41. Lin FC, Chuang YS, Hsieh HM, Lee TC, Chiu KF, Liu CK, et al. Early statin use and the progression of Alzheimer disease: A total population-based case-control study. Medicine (United States). 2015;94(47):e2143.

42. Degerman Gunnarsson M, Ingelsson M, Blennow K, Basun H, Lannfelt L, Kilander L. High tau levels in cerebrospinal fluid predict nursing home placement and rapid progression in Alzheimer's disease. Alzheimer's Research and Therapy. 2016;8(1).

43. Nielsen RE, Lolk A, Valentin JB, Andersen K. Cumulative dosages of antipsychotic drugs are associated with increased mortality rate in patients with Alzheimer's dementia. Acta Psychiatrica Scandinavica. 2016;134(4):314-20.

44. Mueller C, Huntley J, Stubbs B, Sommerlad A, Carvalho AF, Perera G, et al. Associations of Neuropsychiatric Symptoms and Antidepressant Prescription with Survival in Alzheimer's Disease. J Am Med Dir Assoc. 2017;18(12):1076-81.

45. Rhodius-Meester HFM, Liedes H, Koene T, Lemstra AW, Teunissen CE, Barkhof F, et al. Disease-related determinants are associated with mortality in dementia due to Alzheimer's disease. Alzheimer's Research and Therapy. 2018;10(1).

46. Mueller C, Perera G, Hayes RD, Shetty H, Stewart R. Associations of acetylcholinesterase inhibitor treatment with reduced mortality in Alzheimer's disease: A retrospective survival analysis. Age and Ageing. 2018;47(1):88-94.

47. Ku LJE, Li CY, Sun Y. Can Persistence With Cholinesterase Inhibitor Treatment Lower Mortality and Health-Care Costs Among Patients With Alzheimer’s Disease? A Population-Based Study in Taiwan. American Journal of Alzheimer's Disease and other Dementias. 2018;33(2):86-92.

48. Black CM, Fillit H, Xie L, Hu X, Kariburyo MF, Ambegaonkar BM, et al. Economic Burden, Mortality, and Institutionalization in Patients Newly Diagnosed with Alzheimer's Disease. Journal of Alzheimer's Disease. 2018;61(1):185-93.

49. Nielsen RE, Valentin JB, Lolk A, Andersen K. Effects of antipsychotics on secular mortality trends in patients with Alzheimer's disease. Journal of Clinical Psychiatry. 2018;79(3).

50. Giil LM, Aarsland D, Hellton K, Lund A, Heidecke H, Schulze-Forster K, et al. Antibodies to multiple receptors are associated with neuropsychiatric symptoms and mortality in Alzheimer's disease: A longitudinal study. Journal of Alzheimer's Disease. 2018;64(3):761-74.

51. Chu CS, Li WR, Huang KL, Su PY, Lin CH, Lan TH. The use of antipsychotics is associated with lower mortality in patients with Alzheimer’s disease: A nationwide population-based nested case-control study in Taiwan. Journal of Psychopharmacology. 2018;32(11):1182-90.

52. Chen TB, Weng SC, Chou YY, Lee YS, Liang CK, Lin CS, et al. Predictors of Mortality in the Oldest Old Patients with Newly Diagnosed Alzheimer Disease in a Residential Aged Care Facility. Dementia and Geriatric Cognitive Disorders. 2019;48(1-2):93-104.

53. Linna M, Vuoti S, Silander K, Hörhammer I, Halminen O, Mikkola T, et al. Impact of Anti-Dementia Medication on the Risk of Death and Causes of Death in Alzheimer's Disease. Journal of Alzheimer's Disease. 2019;71(4):1297-308.

54. Boumenir A, Cognat E, Sabia S, Hourregue C, Lilamand M, Dugravot A, et al. CSF level of β-amyloid peptide predicts mortality in Alzheimer's disease. Alzheimers Res Ther. 2019;11(1):29.

55. Chen NC, Liang CK, Yin CH, Lin YT, Lee CC, Chen CL. Effects of Socioeconomic Status on Alzheimer Disease Mortality in Taiwan. American Journal of Geriatric Psychiatry. 2020;28(2):205-16.

56. de Sousa OV, Mendes J, Amaral TF. Nutritional and Functional Indicators and Their Association With Mortality Among Older Adults With Alzheimer’s Disease. American Journal of Alzheimer's Disease and other Dementias. 2020;35.

57. Zhang YQ, Wang CF, Xu G, Zhao QH, Xie XY, Cui HL, et al. Mortality of Alzheimer's Disease Patients: A 10-Year Follow-up Pilot Study in Shanghai. Canadian Journal of Neurological Sciences. 2020;47(2):226-30.

58. Wattmo C, Blennow K, Hansson O. Cerebrospinal Fluid Biomarker Levels as Markers for Nursing Home Placement and Survival Time in Alzheimer’s Disease. Current Alzheimer Research. 2021;18(7):573-84.

59. Rajamaki B, Hartikainen S, Tolppanen AM. The effect of comorbidities on survival in persons with Alzheimer's disease: a matched cohort study. BMC geriatrics. 2021;21(1):173.

60. Izquierdo Delgado E, Gutiérrez Ríos R, Andrés Calvo M, Repiso Gento I, Castrillo Sanz A, Rodríguez Herrero R, et al. Nutritional status assessment in Alzheimer disease and its influence on disease progression. Neurologia (Engl Ed). 2021.

61. Liew TM. Neuropsychiatric symptoms in early stage of Alzheimer's and non-Alzheimer's dementia, and the risk of progression to severe dementia. Age and Ageing. 2021;50(5):1709-18.

62. van Loenhoud AC, Groot C, Bocancea DI, Barkhof F, Teunissen C, Scheltens P, et al. Association of Education and Intracranial Volume With Cognitive Trajectories and Mortality Rates Across the Alzheimer Disease Continuum. Neurology. 2022;98(16):e1679-e91.

63. Ono R, Uchida K, Nakatsuka K, Megumi M, Fukuda H. Economic Status and Mortality in Patients with Alzheimer's Disease in Japan: The Longevity Improvement and Fair Evidence Study. Journal of the American Medical Directors Association. 2022;23(1):161-4.

64. Armstrong MJ, Song S, Kurasz AM, Li Z. Predictors of Mortality in Individuals with Dementia in the National Alzheimer's Coordinating Center. Journal of Alzheimer's Disease. 2022;86(4):1935-46.
